# A Prognostic Nomogram Combining Immune-Related Gene Signature and Clinical Factors Predicts Survival in Patients With Lung Adenocarcinoma

**DOI:** 10.3389/fonc.2020.01300

**Published:** 2020-08-06

**Authors:** Congkuan Song, Zixin Guo, Donghu Yu, Yujin Wang, Qingwen Wang, Zhe Dong, Weidong Hu

**Affiliations:** ^1^Department of Thoracic Surgery, Zhongnan Hospital of Wuhan University, Wuhan, China; ^2^Hubei Key Laboratory of Tumor Biological Behaviors & Hubei Cancer Clinical Study Center, Wuhan, China; ^3^Department of Biological Repositories, Zhongnan Hospital of Wuhan University, Wuhan, China

**Keywords:** lung adenocarcinoma, immune related gene, prognosis, signature, nomogram

## Abstract

The existence of tumor heterogeneity and complex carcinogenic mechanisms in lung adenocarcinoma (LUAD) make the most commonly used TNM staging system unable to well-interpret the prognosis of patients. Using transcriptome profiling and clinical data from The Cancer Genome Atlas (TCGA) database, we constructed an immune signature based on a multivariate Cox analysis (stepwise model). We estimated the half-maximal inhibitory concentration (IC50) of chemotherapeutic drugs in patients according to the pRRophetic algorithm. Gene-set variation analysis (GSVA) was used to reveal pathway enrichment between groups. Moreover, immune microenvironment landscape was described by single-sample gene-set enrichment analysis (ssGSEA) and CIBERSORT and systematically correlated with genomic of these patients. A prognostic nomogram combining the immune signature and TNM stage to predict the prognosis was developed by multivariate Cox regression. The novel signature with four immune-related genes (MAL, MS4A1, OAS1, and WFDC2) had good robustness, which can accurately distinguish between high- and low-risk patients. Compared with low-risk patients, high-risk patients with a worse prognosis (5-year OS: 46.5 vs. 59.4%, *p* = 0.002) could benefit more from immunotherapy and the application of common chemotherapeutic agents such as cisplatin and paclitaxel (Wilcoxon test, all *p* < 0.05). There were significant differences in tumor immune microenvironment and metabolic pathways between the two groups. Additionally, the constructed nomogram had reliable predictive performance with the C-index of 0.725 (95% CI = 0.668–0.781) in the development set (*n* = 500), 0.793 (95% CI = 0.728–0.858) in the internal validation set (*n* = 250) and 0.679 (95% CI = 0.644–0.714) in the external validation set (*n* = 442). The corresponding calibration curves also showed good consistency. To sum up, we developed an immune-related gene signature and comprehensively evaluated LUAD immune landscape and metabolic pathways. Effective differentiation of high- and low-risk patients and accurate construction of nomogram would be helpful to the development of individualized treatment strategies.

## Introduction

Lung cancer is the most common malignancy, with morbidity and mortality ranking first in the world, according to global data released by the International Center for Cancer Research in 2020 (1). Lung cancer is divided into non-small cell lung cancer (NSCLC) and small cell lung cancer (SCLC). As the most common subtype of NSCLC (2), lung adenocarcinoma (LUAD) has complex carcinogenic mechanisms and obvious tumor heterogeneity. Due to the continuous improvement in the diagnosis and treatment of LUAD in recent years, especially the rise of immunotherapy, the prognosis of patients has improved significantly. However, the search for new models of diagnosis and treatment to benefit cancer patients has been the focus of oncologists. It is still necessary to further understand the occurrence and progression of LUAD and to identify strong prognostic biomarkers for LUAD.

Immune-related genes have great significance in the immune mechanism and immune function of the body. As we know, cancer is an extremely complex disease involving interactions between tumor and immune system (3). Immunotargeted therapy has played greatly important roles in improving the prognosis of patients with malignant tumors (4, 5). Nevertheless, the treatment can only be applied to some patients, and there are obvious individual differences in the therapeutic effect of this method (6, 7), which further illustrates the existence of tumor heterogeneity and the complexity of carcinogenic mechanisms. The expression of immune-related genes and the density and type of tumor immune infiltrating cells have been widely studied as prognostic biomarkers of lung cancer (8–10). However, the roles of immune-related genes involved in tumor immune microenvironment are still not fully recognized. In this study, a novel immune signature was constructed. We further revealed the differences in the immune microenvironment between high- and low-risk patients and well-predicted the efficacy of chemotherapy and immunotherapy in both groups. In addition, gene-set variation analysis (GSVA) was also used to explore the molecular mechanisms leading to significantly differential prognosis in high- and low-risk patients. Moreover, we developed a nomogram that can accurately predict the prognosis of patients to improve the efficacy of individualized prediction, which may provide a reference for clinicians to formulate more rational treatment strategies.

## Materials and Methods

### Data Source and Preprocessing

The transcriptome profiling data for 535 cases of lung tumor tissue and 59 cases of lung normal tissue were downloaded directly from the Cancer Genome Atlas (TCGA) Genomic Data Commons Data Portal (https://portal.gdc.cancer.gov/, updated until March 05, 2020). The same method was also used to extract the corresponding clinical data (including age, sex, T stage, N stage, TNM stage, survival time, and status). Additionally, RNA expression profiles and clinical information of 443 LUAD patients in the GSE68465 dataset (11) were downloaded from the Gene Expression Omnibus (GEO) database (https://www.ncbi.nlm.nih.gov/geo/).

### Acquisition of Immune-Related Genes

The immune-related gene sets (IMMUNE_RESPONSE and IMMUNE_SYSTEM_PROCESS) were extracted from the Molecular Signatures Database (MSigDB) (https://www.gsea-msigdb.org/gsea/msigdb/index.jsp). There were 332 immune-related genes in these two genomes. To increase the available genes, we also downloaded a total of 2,498 immune-related genes in the Gene List from ImmPort (http://www.immport.org/). After deleting duplicate genes, 1,986 genes were finally used for the next analysis. We obtained immune-related genes and their expression profiles in combination with mRNA gene sets extracted from the TCGA database.

### Differentially Expressed mRNAs (DEMs) in Lung Normal and Tumor Tissues

DEMs between lung normal and tumor tissues were identified by differential expression analysis using the “limma” package in R (12). |log_2_ FC (fold-change)| > 1 and *P* < 0.05 were set as the thresholds for screening DEMs. The common DEMs of the two databases (TCGA and GEO) were used for further analysis.

### GO and KEGG Enrichment Analyses of the Common DEMs

To explore in depth the possible biological processes (BP), cellular components (CC), molecular functions (MF), and pathways of the common DEMs, we carried out GO and KEGG enrichment analysis utilizing the “clusterProfiler” package in R (13) with a statistical threshold of *p* < 0.05.

### Screening of Immune-Related Genes Affecting Prognosis

In order to identify prognosis-related genes, the patients without accurate survival data (e.g., survival time =0 day and unknown) were removed from this study. Finally, 500 patients with detailed survival information were included in the study. Using univariate Cox analysis, we evaluated the association of the common DEMs with OS of LUAD patients. Only these genes with *p* < 0.05 in both two databases (TCGA and GEO) were considered as candidate immune-related genes affecting prognosis.

### Construction and Evaluation of Immune-Related Gene Signature

**Figure 1:**
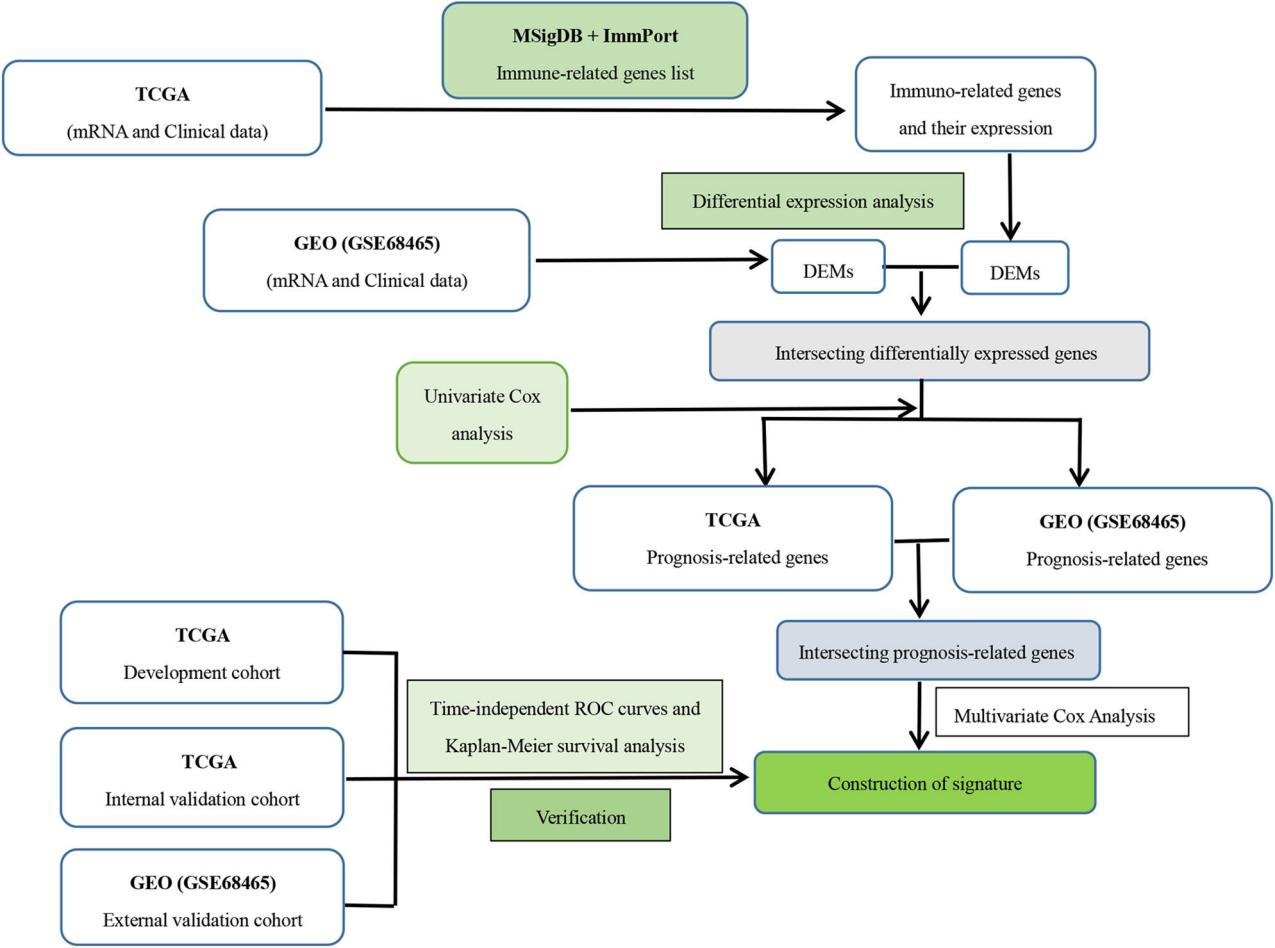
Flowchart on construction and validation of the immune-related gene signature. DEM, differential expressed mRNAs.

Through a multivariate Cox analysis (stepwise model), we filtered these candidate immune-related genes affecting prognosis. Akaike information criterion (AIC) was used to avoid overfitting. We selected the genes with the highest likelihood ratio and lowest AIC values and estimated the β regression coefficients. Based on the β regression coefficients and expression values of the filtered genes, we calculated the risk score of each sample according to the following formula (14):

$$\begin{array}{l} \text{risk}Score=\overset{n}{\underset{i\;=\;1}{\sum }}Coefi\;*\;Expressioni, \end{array}$$

where Coefi was the β regression coefficients obtained from multivariate Cox analysis and Expressioni was the expression of the immune-related genes in the signature. With the median risk score as the cutoff point, patients were divided into high- and low-risk groups. The Kaplan–Meier method was performed to assess the survival differences between the two groups. To further assess the specificity and sensitivity of the immune-related gene-based signature, the ROC curves were drawn and the corresponding AUC values were also calculated. Additionally, we also used the same method to verify the prognostic performance in the internal and external validation datasets. A specific process for constructing this signature is shown in Figure 1.

### Evaluation of the Sensitivity of Chemotherapeutic Agents

To predict the half-maximal inhibitory concentration (IC50) of chemotherapy drugs in the high- and low-risk groups of LUAD patients and to infer the sensitivity of the different patients, we used the “pRRophetic” package in R. By constructing the ridge regression model based on Genomics of Drug Sensitivity in Cancer (GDSC) (www.cancerrxgene.org/) cell line expression spectrum and TCGA gene expression profiles, the package could apply pRRophetic algorithm to predict drug IC50 (15).

### Prediction of Immunotherapy Efficacy

To explore the relationship between the immune signature and immunotherapeutic efficacy, we adopted two computational methods to infer the immunotherapeutic response of LUAD patients at low and high risk. First, we downloaded the mutation data of LUAD from the TCGA database and calculated the tumor mutational burden (TMB) of each sample. The mutation data was divided into two groups by high- and low-risk samples. Second, an online tool named Tumor Immune Dysfunction and Exclusion (TIDE) (http://tide.dfci.harvard.edu) was applied to infer the anti-PD1 and anti-CTLA4 immunotherapeutic response of each sample based on the transcriptome profiles of the TCGA-LUAD cohort (16).

### Exploration of Tumor Immune Landscape

We obtained a set of marker genes related to immune cell types, including different immune cells, immune-related pathways, and functions from Bindea et al. ssGSEA is a feasibility approach, which can apply the characteristics of immune cell population expression to individual cancer samples and can calculate the rank value of each gene according to the expression profile for subsequent statistical analysis (17, 18). In this study, the “GSVA” package in R (19) was utilized with the ssGSEA method. Moreover, the “estimate” package in R was applied to evaluate the immune score, stromal score, and tumor purity of each sample in the high- and low-risk groups.

Moreover, as a wildly proposed computational algorithm, “Cell type Identification By Estimating Relative Subsets Of RNA Transcripts (CIBERSORT)” (20) (https://cibersort.stanford.edu) was also used to predict immune-infiltrating cells of each LUAD sample in our study. The proportion of 22 immune-infiltrating cells in each sample can be obtained by inputting the expression data of the samples. Then, the samples with *p* < 0.05 were selected for further analysis. Human leukocyte antigen (HLA) was also applied to validate the differences between the two groups. In addition, Spearman correlation analysis was used to explore the relationship between four immune-related genes and risk score and immune infiltration.

### Gene-Set Variation Analysis

Using the “GSEABase” package in R, we applied gene-set variation analysis (GSVA) that was predominantly performed on the 50 hallmark pathways described in the MSigDB, where each pathway-related gene set was trimmed to contain only unique genes to reduce pathway overlap and pathway redundancy and most genomes retained 70% of the genes involved (21). MSigDB is a collection of annotated gene sets for use with GSEA software. The MSigDB gene sets are divided into 8 major collections (H, C1–C7). We downloaded “c5.all.v7.0.symbols” (GO gene sets that contain genes annotated by the same GO term). The C5 collection was divided into three subcollections based on GO ontologies: biological process (BP), cellular component (CC), and molecular function (MF). To reveal pathway enrichment between low- and high-risk patients, we used the “GSVA” package in R (19) to evaluate t score and assign pathway activity conditions. Moreover, “limma” package in R was also applied to display distinctions in pathway activation between low- and high-risk groups.

### The Relationship Between Immune-Related Genes and Transcription Factors

We acquired a transcription factor (TF) list from a web application named Cistrome (http://cistrome.org/) and then integrated with the mRNA expression matrix from the TCGA database to derive these TFs' expression level. We examined the correlation between the expression level of the immune-related genes in the signature and each TF using two-sided Pearson correlation coefficients and the *Z*-test. The TFs positively or negatively correlated with the four immune-related genes were considered as immune-related gene-associated TFs (|Pearson correlation coefficients| >0.3 and *P* < 0.001).

### Clinical Correlation and Independent Prognostic Analysis

To better understand the impact of the signature and clinical features on patient outcomes, the univariate and multivariate Cox analyses were performed, which may reveal independent prognostic factors in LUAD patients. In addition, the correlation between the immune-related genes in the signature and clinical features was further explored.

### Construction and Verification of a Prognostic Nomogram

Based on the multivariate Cox analysis, we developed a nomogram for predicting LUAD prognosis in the TCGA database. This nomogram incorporated two predictors, namely, risk score and TNM stage. To further verify the predictive power of this nomogram, we used the 50% LUAD samples randomly selected from the entire TCGA database as internal validation dataset (*n* = 250) and the GSE68465 dataset from the GEO database as external validation dataset (*n* = 442). The C-index and calibration plots were used to assess the performance of the established nomogram.

### Statistical Analysis

All statistical analysis was conducted in R software 3.6.0. All categorical variables were expressed as number (percentage). The Wilcoxon rank-sum test was performed to compare the differences between groups of continuous data. The relationships between immune-related genes and risk score and immune infiltration were determined by the Spearman's correlation analysis. The Kaplan–Meier method was used to survival analysis, where the log-rank test was applied to compare the survival distribution. The Cox proportional hazard model was performed to estimate the β regression coefficient, hazard ratios, *p*-value, and their corresponding 95% confidence interval for each of the selected risk predictors. Based on the multivariate Cox analysis, a nomogram was constructed with the “rms” package in R. The C-index and calibration curve with the bootstrap method were used to evaluate the prediction performance of the nomogram. A *P* < 0.05 was considered statistically significant.

## Results

### The Common DEMs and Functional Annotation

**Figure 2:**
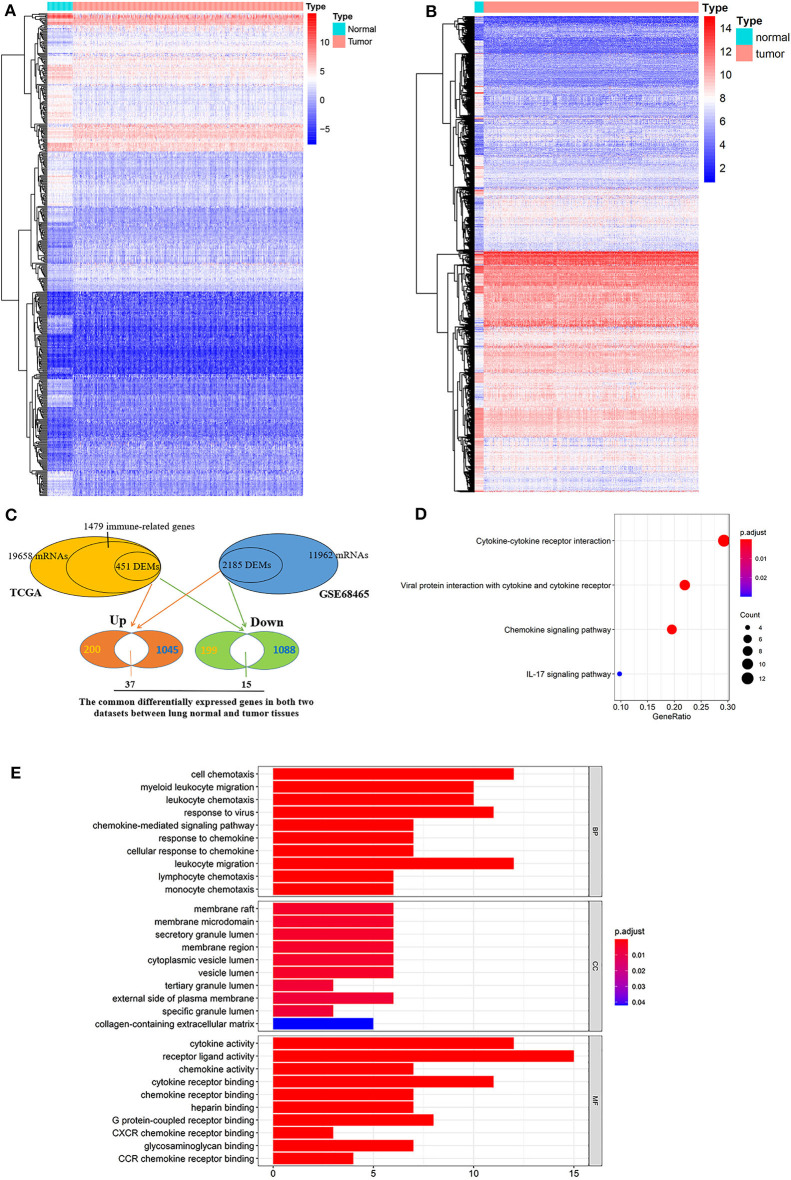
Intersecting genes of differentially expressed genes in two databases and functional enrichment analysis. **(A)** Differential expression analysis of immune-related genes in the TCGA database. **(B)** Differential expression analysis of mRNAs in the GSE68465 dataset. **(C)** Identification of intersection genes for differentially expressed genes in the two datasets. **(D)** KEGG analysis and **(E)** GO enrichment analysis of the common differential expressed genes in two databases.

Of 1,986 immune-related genes obtained from the MSigDB and ImmPort databases, 1,479 genes had corresponding relationships in the TCGA transcriptome. Their expression profiles were used for differential expression analysis (Figure 2A). There were 451 differentially expressed genes in lung tumor and normal tissues, of which 237 were upregulated and 214 were downregulated (Figure 2C). Also, in the GSE68465 dataset, 2,185 genes were differentially expressed in lung tumor and normal tissues (1,082 upregulated genes and 1,103 downregulated genes) (Figures 2B,C).

GO and KEGG enrichment analysis revealed that there were 4 enriched pathways and 361 GO terms (Table S1), of which 4 enrich pathways are shown in Figure 2D and the first 30 GO terms are shown in Figure 2E. KEGG pathway enrichment analysis pointed out that these genes were involved in cytokine–cytokine receptor interaction, viral protein interaction with cytokine and cytokine receptor, chemokine signaling pathway, and IL-17 signaling pathway. Moreover, GO enrichment analysis indicated that these genes were enriched in receptor ligand activity, cell chemotaxis, leukocyte migration, cytokine activity, response to virus, etc.

### Robustness of the Novel Signature Based on Four Immune-Related Genes

**Figure 3:**
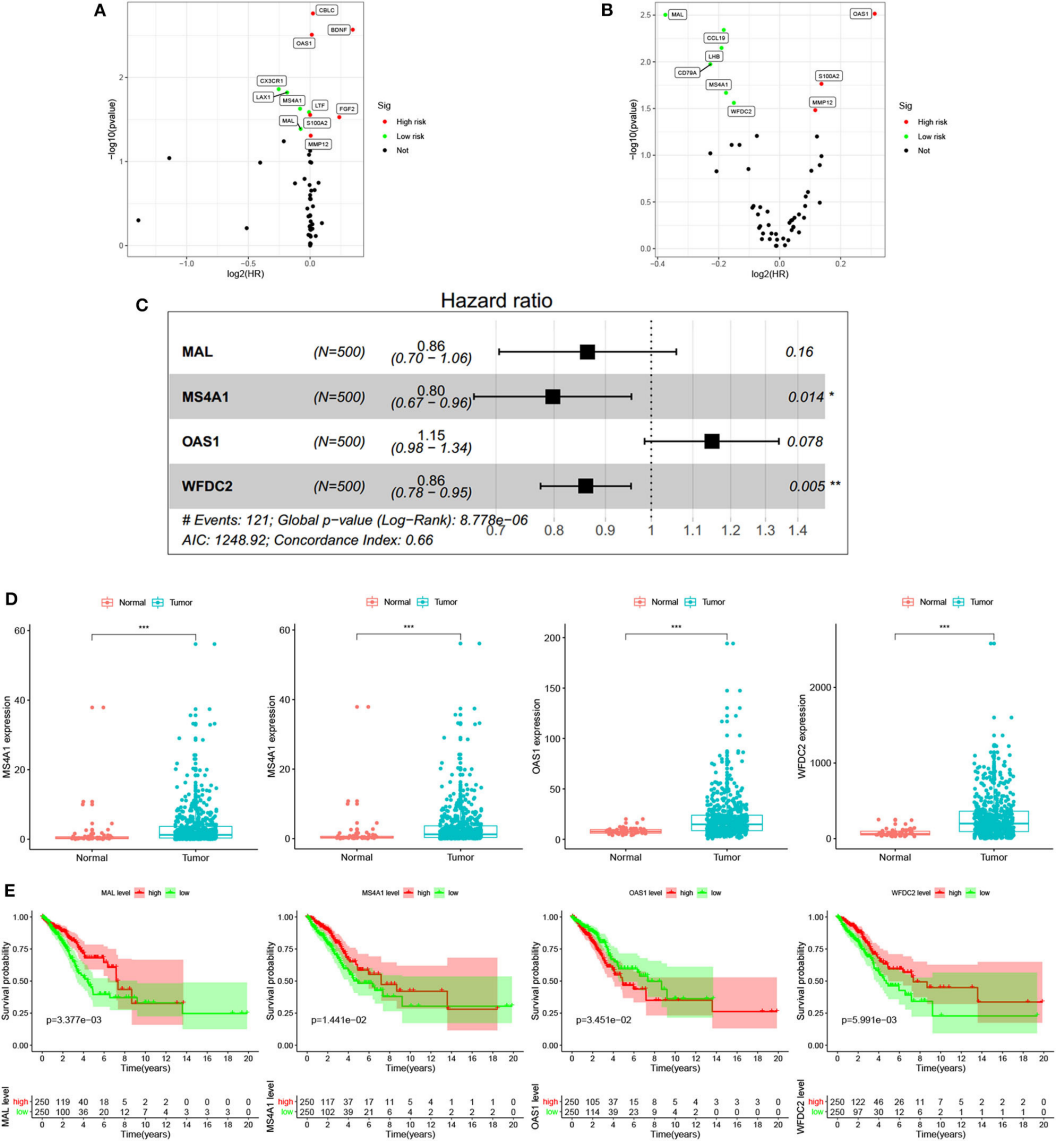
Screening of prognostic immune-related genes and prediction signature construction. Univariate Cox analysis of the common differential expressed genes using the data from the TCGA database **(A)** and using the data from GEO database **(B)**. Each point represents a gene, where the red point represents a high-risk gene whose overexpression is detrimental to patient prognosis, and green dots have the opposite meaning. **(C)** Multivariate Cox analysis of genes with prognostic impact in both datasets using the TCGA database expression matrix and clinical information. **(D)** Differential expression of four immune-related genes (MAL, MS4A1, OAS1, and WFDC2) in the signature between lung normal and tumor tissues. **(E)** Kaplan-Meier survival analysis of four immune-related genes (MAL, MS4A1, OAS1, and WFDC2) in the signature. (*p < 0.05, **p < 0.01, and ***p < 0.001).

**Figure 4:**
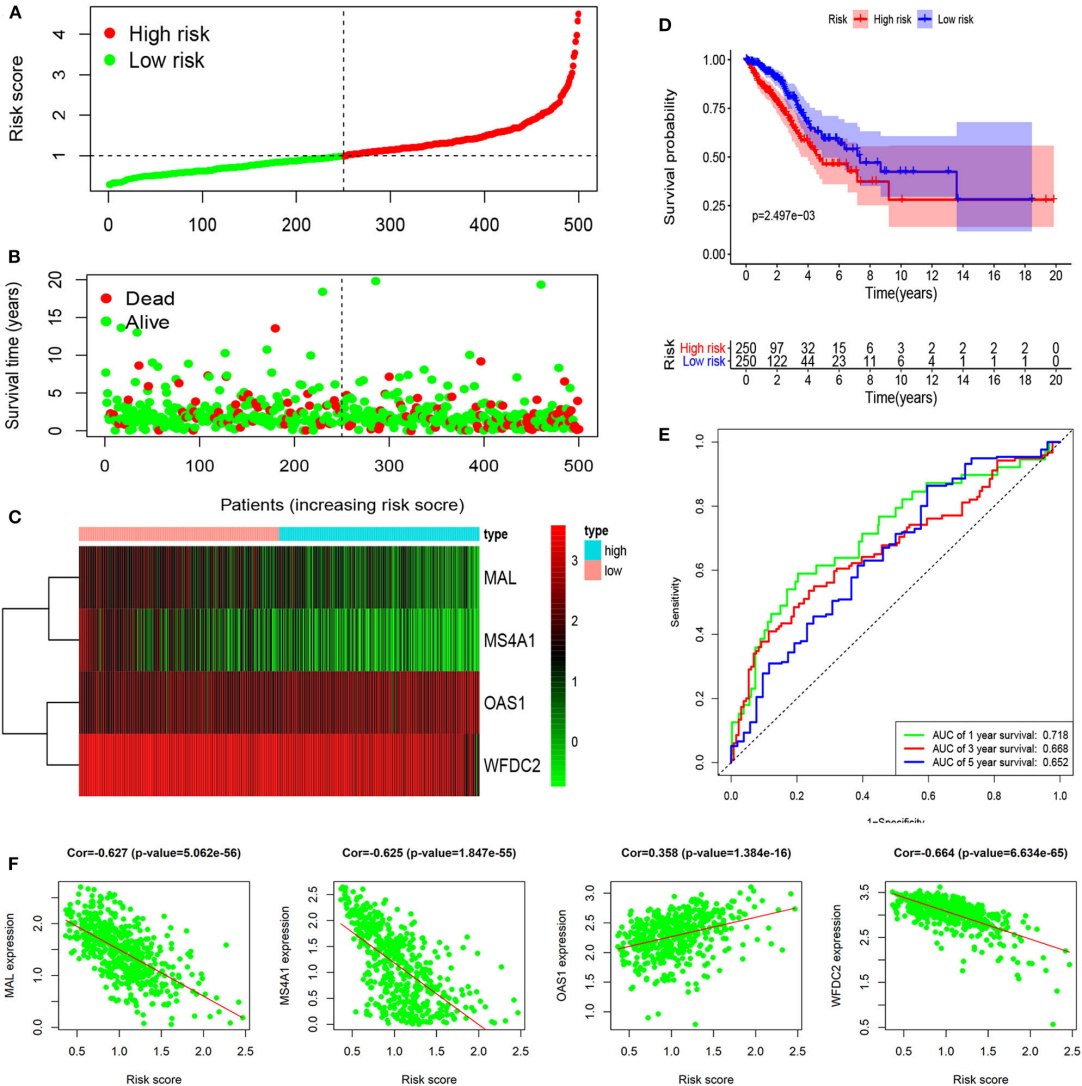
Identification and evaluation of a four-immune-related gene signature to predict OS in the development set. **(A)** The risk score distribution, **(B)** OS status, and **(C)** heat map of the four-immune-related gene signature. **(D)** Kaplan–Meier curves for OS based on the four-immune-related gene signature. The tick marks on the curve represent the censored subjects. The number of patients at risk is listed below the curve. **(E)** The ROC curve analysis of the four-immune-related gene signature for predicting OS. **(F)** Correlation between four immune-related genes and risk score.

Six immune-related genes (CD79A, MAL, MMP12, MS4A1, OAS1, and WFDC2) were identified to significantly influence patient outcomes (all *p* < 0.05) in both the TCGA (Figure 3A) and GSE68465 (Figure 3B) datasets and were included in the multivariate Cox analysis (Table S2). After the multivariate Cox analysis (stepwise models), there were finally four genes (MAL, MS4A1, OAS1, and WFCD2) included in the signature according to their risk coefficients (Figure 3C). Of them, the hazard ratios (HRs) of three genes (MAL, MS4A1, and WFDC2) were <1, indicating that their overexpression was associated with longer OS, while the other gene (OAS1) with HR >1 had the opposite meaning. The expression of these four genes and their relationship to survival are also shown in Figures 3D,E. The constructed risk score formula is shown Risk score = (−0.146 × ExpressionMAL) + (−0.227 × ExpressionMS4A1) + (0.139 × ExpressionOAS1) + (−0.150 × ExpressionWFDC2), through which we estimated the risk score of each patient. Taking the median risk score as the cutoff point, 500 patients were classified into a high-risk group (*n* = 250) and a low-risk group (*n* = 250). The distribution of immune-related genes based on risk score, survival status, and four-gene expression data are shown in Figures 4A–C (development set, *n* = 500), Figures S1A–C (internal validation set, *n* = 250), and Figures S2A–C (external validation set, *n* = 442). The Kaplan–Meier curve analysis in the three datasets obviously demonstrated that patients in the high-risk group had shorter overall survival than those in the low-risk group (log-rank test, all *p* < 0.05; Figure 4D, Figures S1D, S2D). The ROC curves in the development set had a 1-year survival AUC value of 0.718, 3-year survival AUC value of 0.668, and 5-year survival AUC value of 0.652 (Figure 4E). The ROC curves in the internal validation set and external validation set also showed the accuracy of the model in predicting 1-, 3-, and 5-year survival (Figures S1E, S2E). In addition, in three of the four genes (MAL, MS4A1, and WFDC2), their expression value was negatively correlated with the risk score (all cor < −6, all *p* < 0.001), while the other gene (OAS1) was opposite (cor = 0.358, *p* < 0.001). Three datasets also showed the same results (Figure 4F, Figures S1F, S2F).

### Response of High- and Low-Risk Patients to Chemotherapy and Immunotherapy

**Figure 5:**
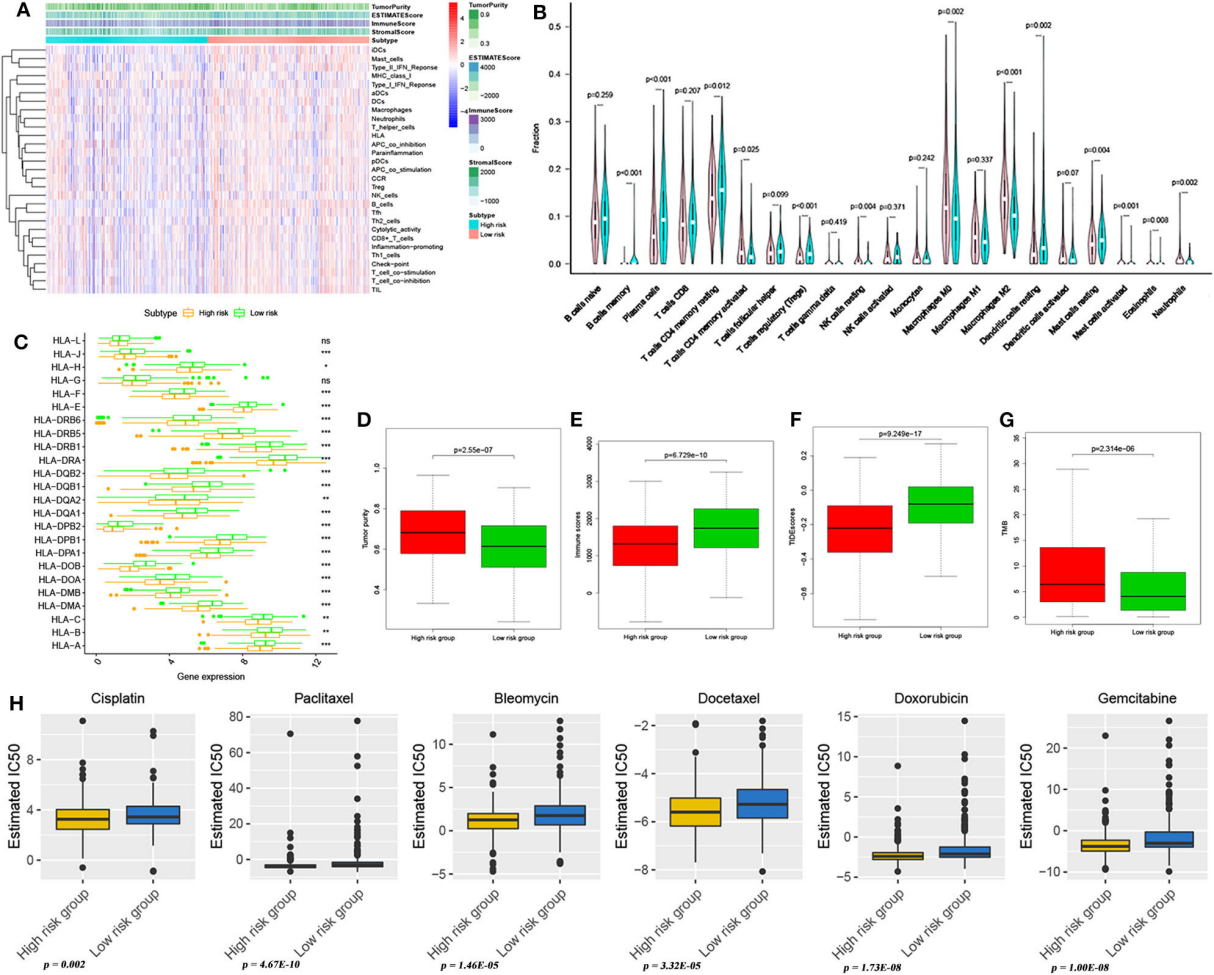
Immune microenvironment landscape and prediction of immunotherapy and chemotherapy effect. **(A)** Immune microenvironment landscape exploration through ssGSEA methods in high- and low-risk patients. **(B)** The comparison of immune infiltration level between high- and low-risk patients, based on CIBERSORT. **(C)** HLA-related gene expression level in high- and low-risk group patients. *p < 0.05, **p < 0.01, and ***p < 0.001. The tumor purity difference **(D)** and immune score difference **(E)** between high- and low-risk patients. Differences in TIDE scores **(F)** and TMB **(G)** between patients in high- and low-risk groups. **(H)** Sensitivity analysis of six common chemotherapeutic drugs in patients at high and low risk.

According to the pRRophetic algorithm, we predicted the IC50 of six common chemotherapeutic agents (cisplatin, bleomycin, docetaxel, doxorubicin, gemcitabine, and paclitaxel) in high- and low-risk patients and found that all six drugs had higher IC50 in low-risk patients (Wilcoxon test, all *p* < 0.05; Figure 5H). It can be indicated that the high-risk patients were more sensitive to these 6 drugs. In addition, using an online tool TIDE program, TIDE scores were calculated to investigate the effectiveness of immune checkpoint (PD-1 and CTLA-4) inhibitors in immunotherapy in two groups. High-risk patients had markedly lower TIDE score compared with low-risk patients (Wilcoxon test, *p* < 0.001; Figure 5F), indicating that high-risk patients may respond better and had better outcome when receiving immune checkpoint (PD-1 and CTLA-4) inhibitors. In addition, the TMB of high- and low-risk patients was investigated in this study. Tumor mutations in both groups are shown in Figure S3. The results showed that high-risk patients had higher TMB than low-risk patients (Wilcoxon test, *p* < 0.001; Figure 5G).

### Differences in Tumor Immune Landscape Between High- and Low-Risk Patients

Comparing the immune infiltration of high- and low-risk groups with two different approaches, we observed that there were significant differences in the components of immune infiltration between the two groups (Figures 5A,B). In the high-risk group, the proportions of iDCs, mast cells, type II IFN response, neutrophils, T helper cells, and inflammatory promoting cells were significantly higher than those of the low-risk group (Wilcoxon test, all *p* < 0.05) (Figure 5A). Similarly, mast cells, eoshophils, neutrophils, and others had higher infiltrations in high-risk groups (Wilcoxon test, all *p* < 0.05) (Figure 5B). Comparing tumor purity and immune score of high- and low-risk patients, we found that LUAD patients in the high-risk group had a lower immune score (Wilcoxon test, *p* < 0.001; Figure 5E) and higher tumor purity (Wilcoxon test, *p* < 0.001; Figure 5D) than patients in the low-risk group. Human leukocyte antigen (HLA) is a major histocompatibility complex (MHC) in humans, closely related to human immune system function, and it also is an important genetic genome of the human immune system. Thus, we further explored the differences in the expression of HLA-related genes between high- and low-risk patients and found that, in addition to HLA-L and HLA-G, the expression levels of other HLA-related genes were significantly different between high- and low-risk groups, that is, these genes had a higher expression in low-risk patients (Wilcoxon test, all *p* < 0.05; Figure 5C). These findings seem to shed light on HLA's important roles in antitumor activity.

We further explored the effects of the four immune-related genes and risk score on the immune infiltration in high- and low-risk patients and found that there was a significant positive correlation between MAL expression level and the infiltration of B cells (cor > 0.4, *p* < 0.001) (Figure S4A) and mast cell resting (cor > 0.2, *p* < 0.01) (Figure S4H). The MS4A1 expression level was significantly positively correlated with B cells (cor > 0.75, *p* < 0.001) (Figure S4B) and B cell memory (cor > 0.4, *p* < 0.001) (Figure S4I) infiltration. OAS1 expression level and type I IFN response (cor > 0.6, *p* < 0.01) (Figure S4D) as well as macrophage M1 (cor > 0.25, *p* < 0.01) infiltration levels (Figure S4E) were significantly positively correlated. In addition, WFCD2 expression level was also found to be significantly correlated with iDCs (cor > 0.3, *p* < 0.01) (Figure S4C) and T cells CD4 memory activated (cor < −0.2, *p* < 0.0025) (Figure S4J) infiltration levels. Moreover, risk score and B cells (cor < −0.5, *p* < 0.004) (Figure S4F) as well as NK cells resting (cor > 0.3, *p* < 0.005) (Figure S4G) infiltration level showed significantly related.

### Differences in Metabolic Pathways Between High- and Low-Risk Patients

**Figure 6:**
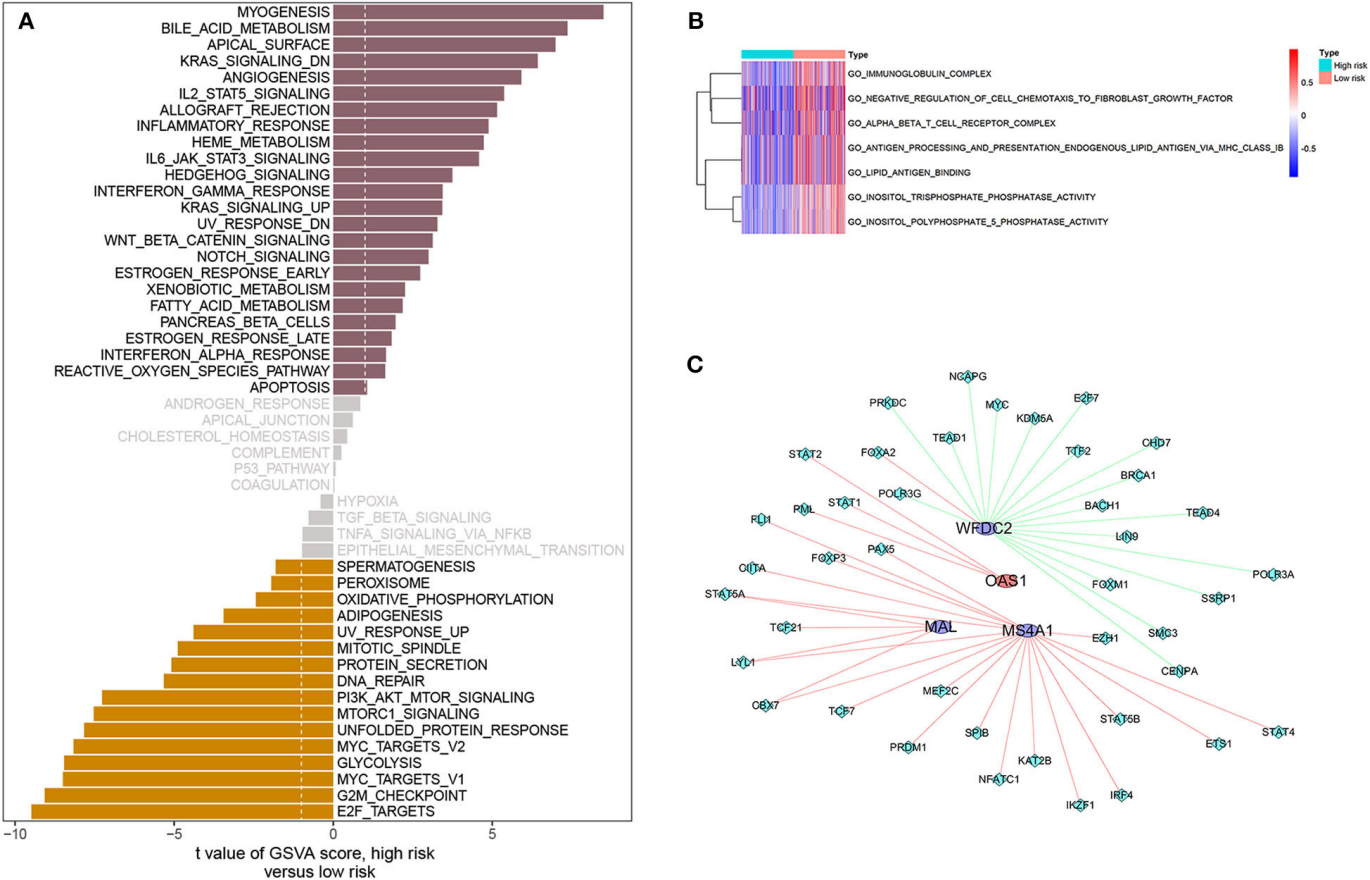
Gene-set variation analysis and correlation between four immune-related genes and TFs. **(A)** Differences in pathway activities scored by GSVA between high- and low-risk patients. T values are shown from a linear model. We set |t| > 1 as a cutoff value. The pink column indicates activated pathways in high-risk patients, and the orange column indicates activated pathways in low-risk patients (DN, down; UV, ultraviolet; v1, version 1; v2, version 2). **(B)** Pathway enrichment analysis based on GO gene sets, including BP, CC, and MF, between high- and low-risk patients. |log2FC| > 0.2 was considered as a cutoff value. Red indicates activated pathways in low-risk patients, and blue indicates activated pathways in high-risk patients. **(C)** Network diagram of four immune-related genes interacting with TFs. The circles represent immune-related genes, where red is for high-risk genes and blue is for low-risk genes. Rhombus represents TFs. Red whip represents positive correlation, and green whip represents negative correlation.

Analysis of hallmark pathway gene signatures indicated that signaling pathways converging at various biological processes were obviously different between high- and low-risk patients. Of note, high-risk patients were more relevant in downregulation of Kras signaling, apical surface, bile acid metabolism, and myogenesis pathways. In comparison, low-risk patients were preferentially related to E2F targets, G2M checkpoint, MYC targets V1, glycolysis, MYC targets V2, unfolded protein response MTORC1 signaling, and PI3K-AKT-MTOR signaling pathways (|log_2_FC| > 0.1, all *p* < 0.001; Figure 6A, Table S3). In addition, GO gene-set variation analysis revealed that phosphatase activity of inositol triphosphate, inositol polyphosphate 5 phosphatase activity, immunoglobulin complexity, and negative regulation of cell-to-fibroblast growth factor chemotaxis of endogenous lipid antigen MHC IB treatment to present lipid antigen binding and alpha beta T cell receptor complex were enriched in the low-risk patients (|log_2_FC| > 0.1, all *p* < 0.001; Figure 6B, Table S3).

### Transcription Factors Linked to Four Immune-Related Genes

Most transcription factors (TFs) are associated with the cell cycle and play a vital role in the induction of proto-oncogene and tumor suppressor gene. We obtained 318 TFs from the Cistrome program (http://cistrome.org/). By co-expression analysis, we finally identified 45 TFs associated with the four immune-related genes (Table S4). Their interrelation is visualized in Figure 6C. Of these four genes, the genes co-expressed with the most TFs were MS4A1 (*n* = 19) and WFDC2 (*n* = 19), the least OAS1 (*n* = 3), which could be seen intuitively from the visual network diagram.

### Relationship Between Clinical Factors and Four Immune-Related Genes as Well as Patient Prognosis

On the basis of the obtained sample clinical characteristics (Table 1), we performed a univariate as well as a multivariate Cox survival analysis. Risk score was identified to be independent prognostic factors for patients with LUAD in both the TCGA database and GSE68465 dataset (all *p* < 0.001; Table 2). Additionally, we also analyzed the correlation between important clinical characteristics and four immune-related genes (Figure S5, Table S5). There were significant correlations between MAL expression and sex (*p* = 0.006; Figure S5A) and T stage (*p* = 0.006; Figure S5B). MS4A1 expression was associated with age (*p* = 0.024; Figure S5C), sex (*p* = 0.001; Figure S5D), lymph-node metastasis (*p* = 0.008; Figure S5E), T stage (*p* < 0.001; Figure S5F), and TNM stage (*p* < 0.001; Figure S5G). In addition, significant correlations were observed between OAS1 expression and lymph-node metastasis (*p* = 0.001; Figure S5H) and TNM stage (*p* = 0.048; Figure S5I). WFDC2 expression was associated with TNM stage (*p* = 0.040; Figure S5J). Specific correlations between the four genes and clinical factors are shown in Table S5. Overall, MAL was expressed higher in women and stage T1&T2 patients. MS4A1 was expressed higher in women and the older (>65 years), stage N0, stage T1&T2, and stage I&II patients, while in the patients with lymph-node metastasis and advanced TNM stage, OAS1 had higher expression. Additionally, WFDC2 higher expression was associated with earlier TNM stages.

**Table 1:** Basic clinicopathologic features.

**Characteristics**	**Subsets**	**TCGA** **development set (*n =* 500)** **(*N*, %)**	**TCGA** **internal validation set (*n =* 250)** **(*N*, %)**	**GEO** **external validation set (*n =* 442)** **(*N*, %)**
Age (years)	<65	219 (43.8)	115 (46.0)	214 (48.3)
	≥65	271 (54.2)	129 (51.6)	229 (51.7)
	Unknown	10 (2.0)	6 (0.24)	0 (0.00)
Sex	Female	270 (54.0)	122 (48.8)	220 (49.6)
	Male	230 (46.0)	128 (51.2)	223 (50.4)
T stage	T1&T2	434 (86.8)	218 (87.2)	401 (90.6)
	T3&T4	63 (12.6)	31 (12.4)	40 (9.0)
	Tx	3 (0.6)	1 (0.4)	2 (0.4)
N stage	N0	324 (64.8)	157 (62.8)	229 (67.5)
	N1&N2&N3	165 (33.0)	87 (34.8)	141 (31.9)
	Nx	11 (2.2)	6 (2.4)	3 (0.6)
TNM stage	I&II	387 (77.4)	190 (76.0)	371 (83.8)
	III&IV	105 (21.0)	54 (21.6)	69 (15.6)
	Unknown	8 (1.6)	6 (2.4)	3 (0.6)
Risk score	High risk	250 (50.0)	124 (49.6)	221 (50.0)
	Low risk	250 (50.0)	126 (50.4)	221 (50.0)

**Table 2:** Univariate and multivariate Cox analysis of the four-immune-related gene signature and clinical risk factors.

**Variables**	**Univariate analysis**		**Multivariate analysis**	
	**HR (95% CI)**	***P*-value**	**HR (95% CI)**	***P*-value**
**TCGA**				
Age (years)	1.012 (0.993–1.032)	0.213	1.020 (1.001–1.040)	0.044
Sex (male vs. female)	0.916 (0.636–1.321)	0.639	0.766 (0.525–1.118)	0.167
T stage (T3&T4 vs. T1&T2)	2.119 (1.315–3.414)	0.002	1.151 (0.669–1.981)	0.612
N stage (N1&N2&N3 vs. N0)	3.161 (2.186–4.569)	<0.001	2.096 (1.354–3.266)	0.001
TNM stage (III&IV vs. I&II)	3.054 (2.099–4.445)	<0.001	1.747 (1.073–2.846)	0.025
Risk score	2.029 (1.654–2.489)	<0.001	1.921 (1.526–2.418)	<0.001
**GSE68465**				
Age (years)	1.017 (1.004–1.031)	0.013	1.018 (1.004–1.032)	0.010
Sex (male vs. female)	1.224 (0.946–1.584)	0.124	1.051 (0.808–1.368)	0.712
T stage (T3&T4 vs. T1&T2)	1.521 (1.036–2.231)	0.032	1.371 (0.860–2.186)	0.185
N stage (N1&N2&N3 vs. N0)	1.441 (1.107–1.866)	0.007	1.445 (1.057–1.977)	0.021
TNM stage (III&IV vs. I&II)	1.362 (0.983–1.885)	0.063	0.926 (0.588–1.460)	0.742
Risk score	208.6 (21.04–2068.4)	<0.001	192.3 (18.00–2053.7)	<0.001

### Predictive Performance of the Established Nomogram

**Figure 7:**
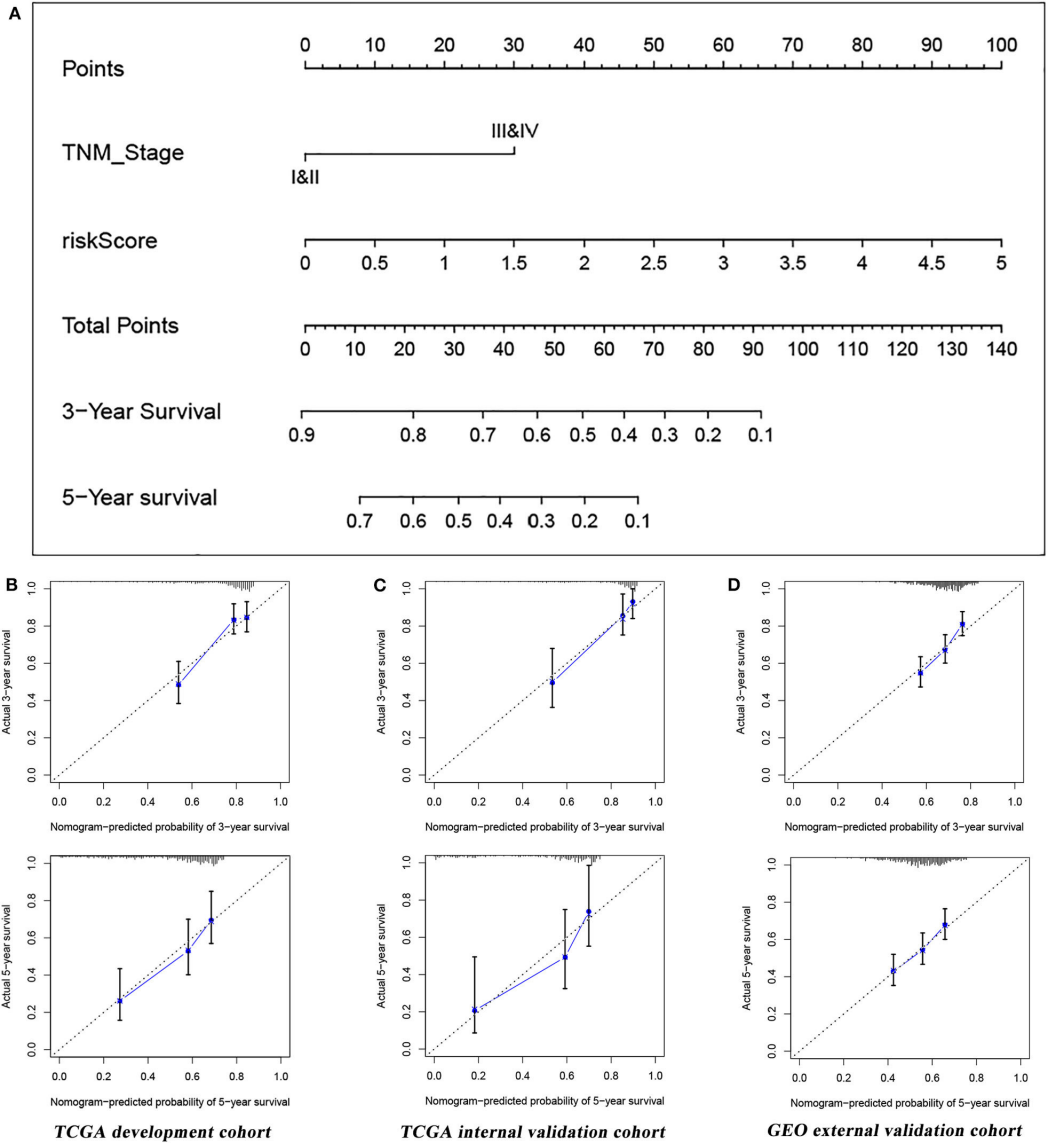
**(A)** A nomogram combining TNM stage and the four-immune-related gene signature for predicting LUAD prognosis. **(B)** Calibration curves of the nomogram for the probability of OS at 3 and 5 years in the TCGA development set. **(C)** Calibration curves of the nomogram for the probability of OS at 3 and 5 years in the TCGA internal validation set. **(D)** Calibration curves of the nomogram for the probability of OS at 3 and 5 years in the GEO external validation set.

Based on the four-immune-related gene signature (risk score) and clinical factor (TNM stage), we constructed a nomogram to predict patients' prognosis in the TCGA database (Figure 7A). According to the multivariate Cox analysis, each factor (in the nomogram) was assigned a score, then the total nomogram score was obtained from the sum of individual scores of all predictors. In association with the total score, 3- and 5-year survival of patients can be estimated by projecting the total points downward. In the present study, we used a bootstrap method to verify the developed nomogram with the C-index of 0.725 (95% CI = 0.668–0.781) in the development set (*n* = 500), 0.793 (95% CI = 0.728–0.858) in the internal validation set (*n* = 250), and 0.679 (95% CI = 0.644–0.714) in the external validation set (*n* = 442), which suggested that the predictive model had good predictive performance. Furthermore, the calibration curves in three datasets also showed good consistency compared with the ideal model, further indicating that the nomogram was stable in predicting the prognosis of LUAD patients (Figures 7B–D).

## Discussion

As one of the malignancies with high morbidity and mortality, lung cancer is a public health concern (22, 23). Due to tumor heterogeneity and complex oncogenic mechanisms in LUAD, it is extremely challenging to develop individualized treatment strategies and accurately predict patient prognosis (24, 25). Increasing researches (26–29) have proved that the prognosis of cancer patients was closely related to tumor microenvironment. Immune responses in tumor microenvironments are also considered important determinants of tumor invasiveness and progression. This study constructed a novel immune signature with good robustness, which could accurately distinguish high- and low-risk patients. On this basis, this study explored the tumor immune microenvironment in high- and low-risk patients and revealed that the high-risk patients had higher tumor purity and lower immune score. Tumor purity and immune score were considered to be important factors affecting the prognosis of cancer patients (30–32). Tumor purity refers to the percentage of tumor cells in the tumor immune microenvironment. Some studies have reported that poor prognosis was closely related to low tumor purity in glioma (30) and colorectal cancer (31). Contrary to the poorer prognosis of low tumor purity in glioma and colorectal cancer, Wang et al. (32) observed that patients with low LUAD purity tended to have a better prognosis. This finding was in line with that of our study. Low tumor purity was associated with different outcomes in different cancer patients, which seemed to indicate that the patterns of occurrence and progression of different tumors were also quite different. In our study, high-risk patients with high tumor purity had poor prognosis. We believe that the survival difference between high- and low-risk patients might be due to higher frequency mutations in key pathways and changes in the tumor microenvironment associated with tumor purity.

Additionally, all kinds of cells, cytokines, and chemokines that interact with tumor cells in the tumor microenvironment, especially immune cells, are increasingly recognized as important roles in the body against tumors. Immune score, which can promote the quantification of immune components (such as immune cells) in tumors, can significantly affect the prognosis of patients. In several studies (32–35), it has been confirmed that high immune scores were associated with better prognosis. Similarly, high-risk patients with worse prognosis had lower immune scores in our study, which further suggested the validity and accuracy of the signature constructed in this study in identifying high-risk patients. Besides, high-risk patients were significantly different from low-risk patients in terms of immune infiltrating cell types, for example iDCs, mast cells, type II IFN response, neutrophils, T helper cells, macrophages M1, and inflammatory promoting cells. Four immune-related genes (MAL, MS4A1, OAS1, and WFDC2) in the signature were also associated with the immune infiltration. As explained by Aran et al. (36), we also thought that the inflammatory response caused by immune cells might promote the mutation of tumor cells, which in turn affected the prognosis of patients. Therefore, it is still an important part of the future research on LUAD to explore the specific mechanism of tumor immune microenvironment on prognosis.

Since the patients with refractory malignant tumors, including lung cancer (4, 5), benefit significantly from immune checkpoint inhibitors, immunotherapy is becoming a new therapeutic option for cancer patients. TMB and immune checkpoint levels (e.g., PD-1, PD-L1, CTLA4) are considered biomarkers for predicting the efficacy of immunotherapy. As a marker for evaluating the effectiveness of immunotherapy (37), the effect of TMB has been confirmed in the treatment of malignant tumors with mismatch repair defects by the PD-1/PD-L1 antibody (38, 39). TIDE is a completely new computational framework designed by Jiang et al. (16), which can integrate the two immune escape mechanisms of tumor (immune dysfunction and rejection) and is believed to be a substitute for a single biomarker to effectively predict the efficacy of immune checkpoint inhibitors. In view of this, this study assessed the effect of immunotherapy of high- and low-risk patients from two aspects (TMB and TIDE scores) and speculated that high-risk patients may benefit more from immunotherapy. It is worth noting that although immunotherapy can bring good benefits to some lung cancer patients, some patients still do not show the desired results after using immune checkpoint inhibitors (6, 7). The current bottleneck in the treatment of lung cancer also makes such patients have to return to traditional chemotherapy to improve the prognosis. Thus, the mRNA expression data from the TCGA database was used to explore the sensitivity of patients at high and low risk to traditional chemotherapeutic agents (cisplatin, bleomycin, docetaxel, doxorubicin, gemcitabine, and paclitaxel). This study indicated that using the same drugs, the high-risk patients may perform better than the low-risk patients. Currently, platinum-based chemotherapy is the standard regimen for the treatment of advanced LUAD. However, the mechanism of drug resistance and the existence of heterogeneity also make the effect of drug therapy unsatisfactory. This study revealed the sensitivity of high- and low-risk patients to six chemotherapeutic drugs including cisplatin, which would provide a visual field for researchers to develop drugs with high therapeutic index or high efficacy.

In addition, this study found that there were significant differences in metabolic pathways between high- and low-risk patients, such as immunoglobulin complexity and negative regulation of cell-to-fibroblast growth factor chemotaxis of endogenous lipid antigen MHC IB treatment to present lipid antigen binding and alpha beta T cell receptor complex. This indicated that the pathogenesis of LUAD was a complex biological process driven by specific gene and epigenetic changes. Moreover, abnormal regulation of multiple genes can promote the occurrence and development of LUAD through different mechanisms. Differences in metabolic pathways between high- and low-risk patients based on the established signature have not been previously reported in LUAD, indicating that the four immune-related genes in the signature and varied gene sets in GSVA between high- and low-risk patients have the potential to be further investigated for deeper analysis. In general, these findings may provide a new perspective for researchers and clinicians in finding breakthroughs in further molecular mechanism studies.

For clinical application, good biomarkers should be those that can accurately predict prognosis for patients, distinguish patients with different risks, and thus assist clinicians to make the most reasonable treatment plan in time. Nomogram may be a good choice for this purpose. Nomogram, a visual statistical tool, was wildly used in prognostic assessment of cancer patients (40, 41). In this study, combining the immune signature and TNM stage, a prognosis nomogram with excellent performance was constructed. This nomogram incorporated two important predictors (risk score and TNM stage). The TNM stage of patients may be easy to obtain, while the acquisition of another predictor (risk score) required knowledge of the expression of four immune-related genes (MAL, MS4A1, OAS1, and WFDC2) in tumor tissues, which undoubtedly increased the burden of nomogram application. This appears to be a common problem for most molecular diagnostic or prognostic models. How to simplify the clinical application of predictive models is a question for researchers and clinicians to consider. We believe that the development of molecular detection technology in the future is bound to improve this dilemma. The nomogram may be used routinely in the future.

The four immune-related genes in the signature have previously been shown to be potential biomarkers. Relevant researches have reported that deletion of MAL gene expression was associated with the development and progression of many malignancies in humans, such as cervical cancer, ovarian cancer, oral cancer, laryngeal cancer, breast cancer, esophageal cancer, gastric cancer, bowel cancer, and renal cancer (42–49). MS4A1, also known as CD20, is a member of the MS4A gene family. MS4A1 (CD20) is an important marker of B cell differentiation and an important target for immunotherapy in lymphoma (50). Anti-CD20 monoclonal antibody (rituximab) became the first monoclonal antibody approved for cancer treatment in 1997, and it could kill tumor cells by complement-dependent and antibody-dependent cytotoxicity. Along with the development of antibody humanization and Fc segment modification, the therapeutic spectrum is not only limited to lymphoma but also includes chronic lymphoblastic leukemia, acute lymphoblastic leukemia, solid tumor, and immune-related diseases (51–54). OAS1 is an important component of the immune system and has significant antiviral effects. It is worth noting that the relationship between these three genes and LUAD is hardly reported in the literature; however, this study found that all three genes were associated with prognosis in patients with LUAD, and there were significant differences in gene expression in patients with different clinical features. In combination with the relationship between these three genes and other tumors and the findings of this study, we believe that these three immune-related genes could affect the immune microenvironment of LUAD and might be involved in the occurrence and progression of LUAD. Additionally, along with further exploration, researchers found that WFDC2 presented a high expression state in lung cancer (55–58) and recognized that WFDC2 as a serum tumor marker had important clinical application in the early diagnosis of lung cancer and the monitoring of a curative effect (59). Nevertheless, the expression of WFDC2 in LUAD and its relationship with patients prognosis were rarely reported. This study found that WFDC2 was significantly associated with TNM stage and prognosis in LUAD patients. In general, these four immune-related genes may play key roles in the development of LUAD. This study provided preliminary evidence that these genes were closely related to the clinical features and prognosis of LUAD, which would provide new research directions and ideas for finding new gene therapy targets and developing new antitumor drugs.

There are some limitations in this study. First, although the signature and nomogram constructed in this study using massive data from TCGA and GEO databases have reliable robustness, the nature of retrospective analysis still exists. Second, we explored the immune microenvironment landscape and molecular mechanisms in patients at different risks and predicted their effects of immunotherapy and chemotherapy, but the study still lacked experimental verification.

To make a long story short, this study identified and validated a novel immune-related gene signature comprising four genes (MAL, MS4A1, OAS1, and WFDC2) in LUAD patients, which can serve as a prognostic predictor of LUAD patients. Additionally, the signature can indicate the sensitivity of LUAD patients to chemotherapeutic agents (cisplatin, bleomycin, docetaxel, doxorubicin, gemcitabine, and paclitaxel) as well as immune checkpoint inhibitors and provide new clinical applications for LUAD patients. Furthermore, the established nomogram with good robustness can accurately predict the prognosis of LUAD patients, which may help doctors make more rational treatment decisions.

## Data Availability Statement

The datasets presented in this study can be found in online repositories. The names of the repository/repositories and accession number(s) can be found in the article/Supplementary Material.

## Ethics Statement

The study was approved by the Institutional Research Committee of Zhongnan Hospital of Wuhan University. Data of our present study was downloaded from the open databases TCGA and GEO, so there was no informed consent from participants. Written informed consent for participation was not required for this study in accordance with the national legislation and the institutional requirements.

## Author Contributions

CS, WH, and ZD designed the study. CS analyzed the data and prepared the manuscript. All authors were substantially involved in the research, acquisition of data, analysis, manuscript preparation, and read and approved the final manuscript.

## Conflict of Interest

The authors declare that the research was conducted in the absence of any commercial or financial relationships that could be construed as a potential conflict of interest.

## Supplementary Material

Figure S1 Identification and evaluation of a four-immune-related-gene signature to predict OS in the TCGA internal validation set. **(A)** The risk score distribution, **(B)** OS status and **(C)** heatmap of the four-immune-related-gene signature. **(D)** Kaplan-Meier curves for OS based on the four-immune-related-gen signature. The tick-marks on the curve represent the censored subjects. The number of patients at risk is listed below the curve. **(E)** The ROC curve analysis of the four-immune-related-gene signature for predicting OS. **(F)** Correlation between four immune-related genes and risk scores.

Figure S2 Identification and evaluation of a four-immune-related-gene signature to predict OS in the GEO external validation set. **(A)** The risk score distribution, **(B)** OS status and **(C)** heatmap of the four-immune-related-gene signature. **(D)** Kaplan-Meier curves for OS based on the four-immune-related-gen signature. The tick-marks on the curve represent the censored subjects. The number of patients at risk is listed below the curve. **(E)** The ROC curve analysis of the four-immune-related-gene signature for predicting OS. **(F)** Correlation between four immune-related genes and risk scores.

Figure S3 Tumor mutations in LUAD patients in TCGA database.

Figure S4 Correlation of four immune-related genes and risk scores with immune-infiltrating cells. Correlation between MAL **(A)**, MS4A1 **(B)**, WFDC2 **(C)**, OAS1 **(D)**, risk score **(F)** and immune infiltration, based on the ssGSEA approach. Correlation between OAS1 **(E)**, risk score **(G)**, MAL **(H)**, MS4A1 **(I)**, WFDC2 **(J)** and immune-infiltrating cells, based on CIBERSORT.

Figure S5 Correlation analysis between four immune-related genes and clinical features. There were significant correlations between MAL expression and sex **(A)** and T stage **(B)**. MS4A1 expression was associated with age **(C)**, sex **(D)**, lymph-node metastasis **(E)**, T stage **(F)**, and TNM stage **(G)**. In addition, significant correlations were observed between OAS1 expression and lymph-node metastasis **(H)** and TNM stage **(I)**. WFDC2 expression was associated with TNM stage **(J)**.

Table S1 GO and KEGG enrichment analysis on the common differentially expressed gene.

Table S2 Univariate Cox analysis of intersection differentially expressed gene in the TCGA and GEO databases.

Table S3 Gene set variation analysis in the high- and low-risk groups.

Table S4 45 transcription factors associated with the four immune-related genes.

Table S5 The relationship between four immune-related genes and clinical features.

## Abbreviations

LUAD: Lung adenocarcinoma
OS: Overall survival
TCGA: The Cancer Genome Atlas
GEO: Gene Expression Omnibus
CI: Confidence interval
HR: Hazard ratio
ROC: Receiver operating characteristic
AUC: Area under the curve
C-index: Concordance index
CTLA-4: Cytotoxic T-lymphocyte associated 4
PD-1: Programmed cell death 1
PD-L1: Programmed cell death-ligand 1
TMB: Tumor mutational burden
GSVA: Gene-set variation analysis
GSEA: Gene-set enrichment analysis.
