# Supplementary figures and images for: A Prognostic Nomogram Combining Immune-Related Gene Signature and Clinical Factors Predicts Survival in Patients With Lung Adenocarcinoma
Source: Front Oncol. 2020 Aug 6;10:1300. doi: 10.3389/fonc.2020.01300 (PMC7424034; doi:10.3389/fonc.2020.01300)

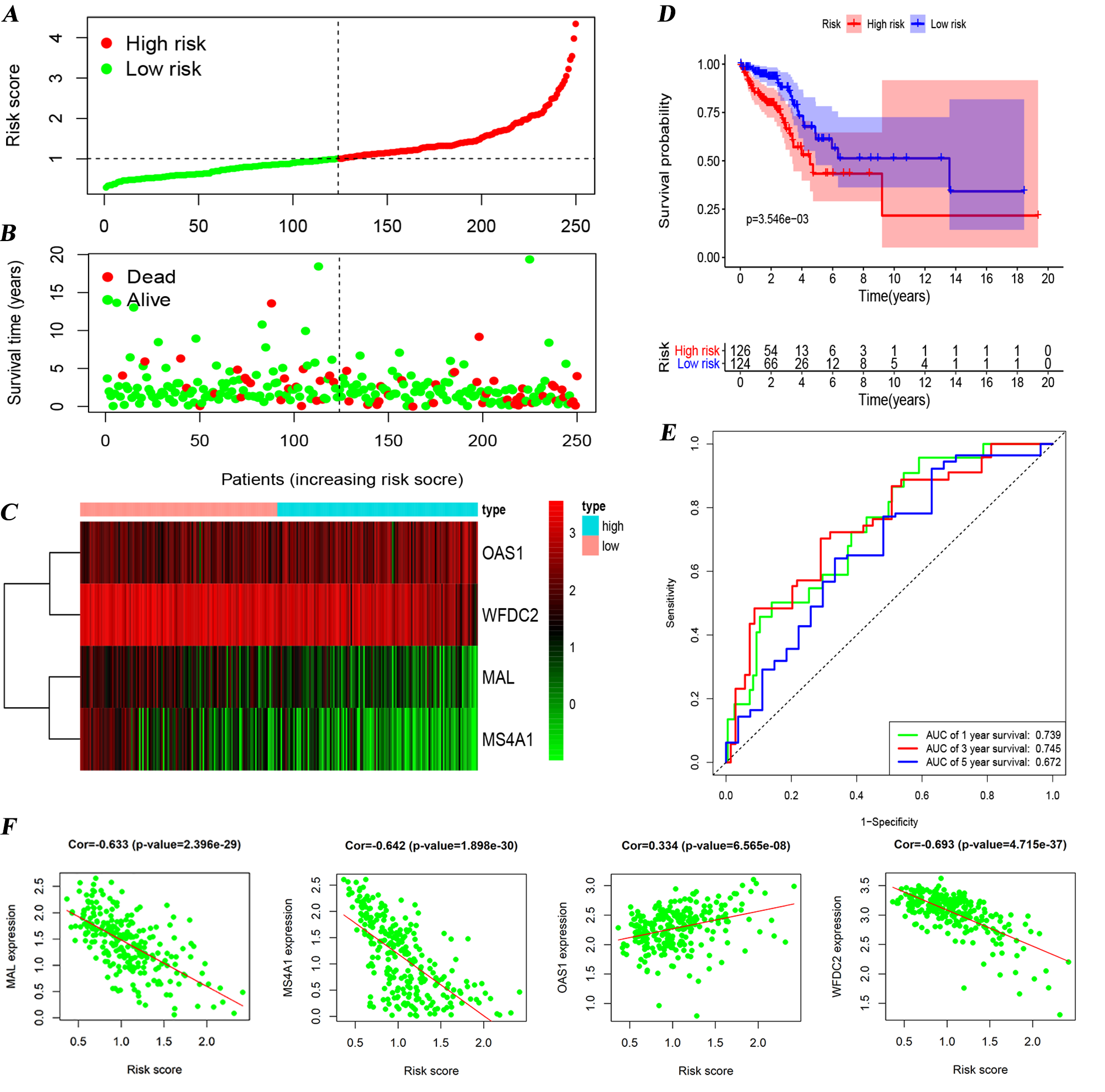

Supplement: Figure S1 — Identification and evaluation of a four-immune-related-gene signature to predict OS in the TCGA internal validation set. (A) The risk score distribution, (B) OS status and (C) heatmap of the four-immune-related-gene signature. (D) Kaplan-Meier curves for OS based on the four-immune-related-gen signature. The tick-marks on the curve represent the censored subjects. The number of patients at risk is listed below the curve. (E) The ROC curve analysis of the four-immune-related-gene signature for predicting OS. (F) Correlation between four immune-related genes and risk scores. [file Image_1.TIF]

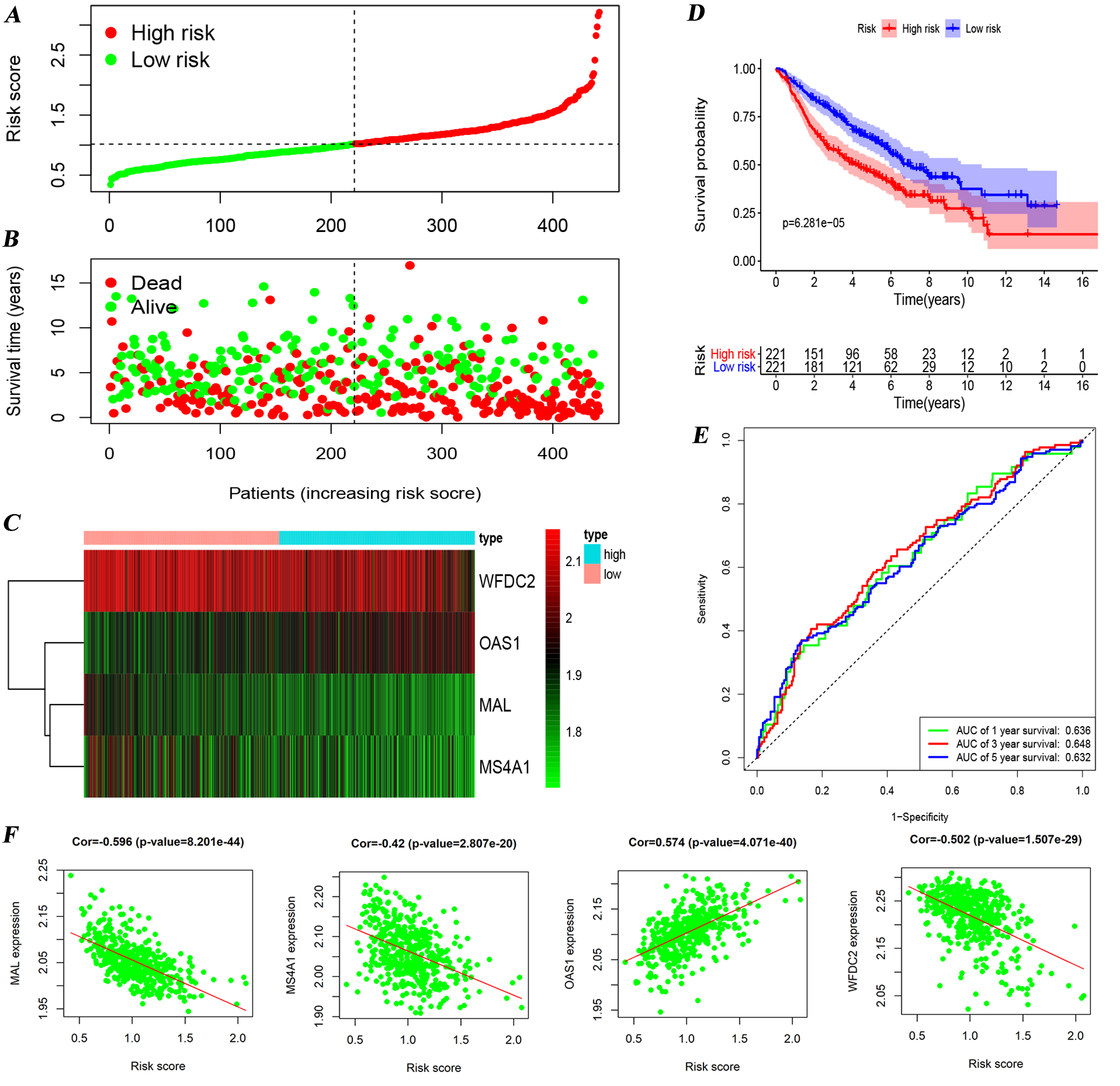

Supplement: Figure S2 — Identification and evaluation of a four-immune-related-gene signature to predict OS in the GEO external validation set. (A) The risk score distribution, (B) OS status and (C) heatmap of the four-immune-related-gene signature. (D) Kaplan-Meier curves for OS based on the four-immune-related-gen signature. The tick-marks on the curve represent the censored subjects. The number of patients at risk is listed below the curve. (E) The ROC curve analysis of the four-immune-related-gene signature for predicting OS. (F) Correlation between four immune-related genes and risk scores. [file Image_2.TIF]

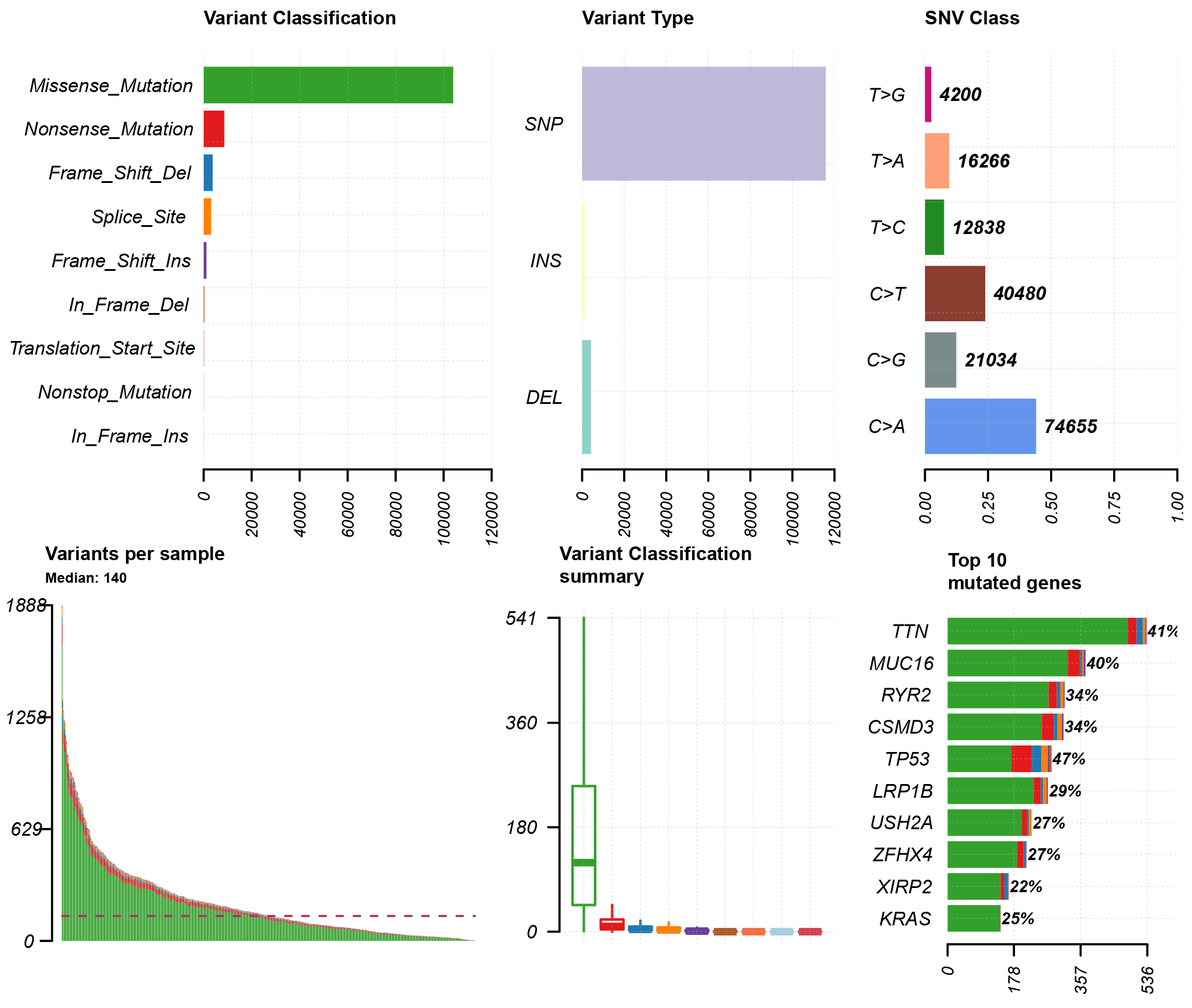

Supplement: Figure S3 — Tumor mutations in LUAD patients in TCGA database. [file Image_3.TIF]

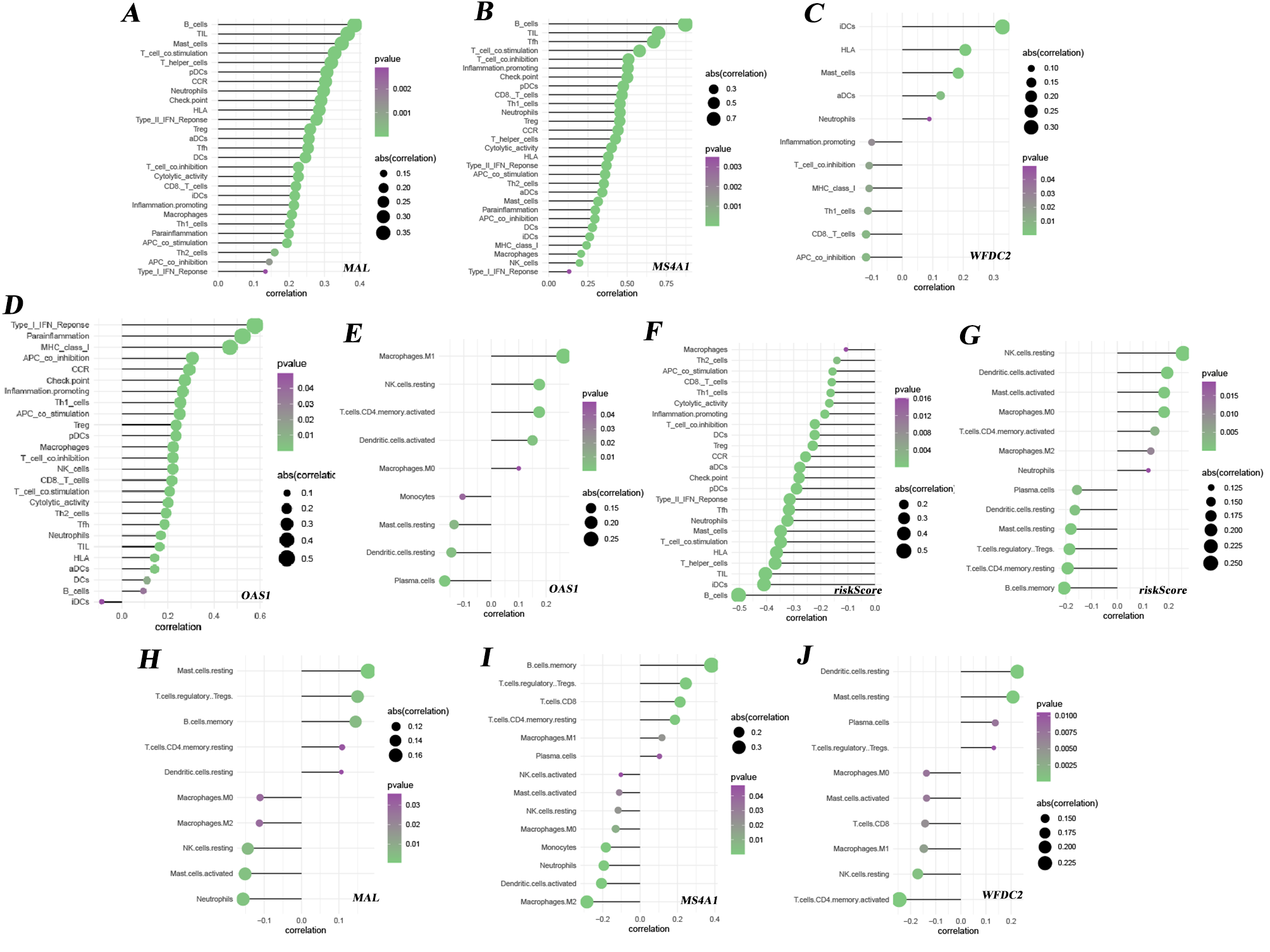

Supplement: Figure S4 — Correlation of four immune-related genes and risk scores with immune-infiltrating cells. Correlation between MAL (A), MS4A1 (B), WFDC2 (C), OAS1 (D), risk score (F) and immune infiltration, based on the ssGSEA approach. Correlation between OAS1 (E), risk score (G), MAL (H), MS4A1 (I), WFDC2 (J) and immune-infiltrating cells, based on CIBERSORT. [file Image_4.TIF]

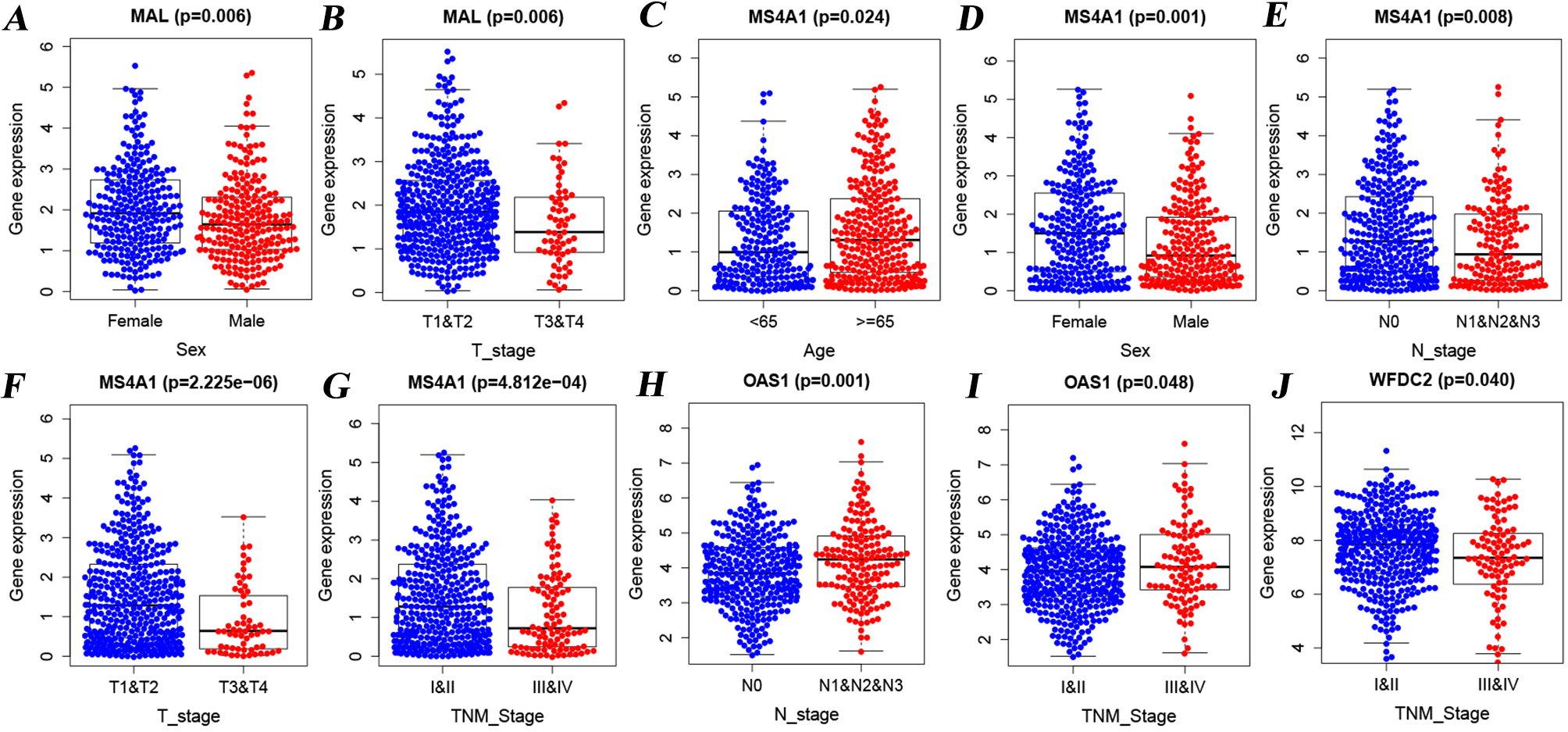

Supplement: Figure S5 — Correlation analysis between four immune-related genes and clinical features. There were significant correlations between MAL expression and sex (A) and T stage (B). MS4A1 expression was associated with age (C), sex (D), lymph-node metastasis (E), T stage (F), and TNM stage (G). In addition, significant correlations were observed between OAS1 expression and lymph-node metastasis (H) and TNM stage (I). WFDC2 expression was associated with TNM stage (J). [file Image_5.TIF]
