# Supplementary material for: A Prognostic Nomogram Combining Immune-Related Gene Signature and Clinical Factors Predicts Survival in Patients With Lung Adenocarcinoma
Source: Front Oncol. 2020 Aug 6;10:1300. doi: 10.3389/fonc.2020.01300 (PMC7424034; doi:10.3389/fonc.2020.01300)
Supplement: Table S1 — GO and KEGG enrichment analysis on the common differentially expressed gene. [file Table_1.DOCX]

**Table S1: GO and KEGG enrichment analysis on the common differentially expressed gene.**

| ID | Description | p.value | p.adjust | Count |
| --- | --- | --- | --- | --- |
| GO:0060326 | cell chemotaxis | 3.23E-11 | 5.34E-08 | 12 |
| GO:0097529 | myeloid leukocyte migration | 2.74E-10 | 2.27E-07 | 10 |
| GO:0030595 | leukocyte chemotaxis | 5.15E-10 | 2.84E-07 | 10 |
| GO:0009615 | response to virus | 1.24E-09 | 5.13E-07 | 11 |
| GO:0070098 | chemokine-mediated signaling pathway | 4.57E-09 | 1.51E-06 | 7 |
| GO:1990868 | response to chemokine | 9.07E-09 | 1.88E-06 | 7 |
| GO:1990869 | cellular response to chemokine | 9.07E-09 | 1.88E-06 | 7 |
| GO:0050900 | leukocyte migration | 9.10E-09 | 1.88E-06 | 12 |
| GO:0055074 | calcium ion homeostasis | 5.57E-08 | 8.38E-06 | 11 |
| GO:0071222 | cellular response to lipopolysaccharide | 9.17E-08 | 1.26E-05 | 8 |
| GO:0071219 | cellular response to molecule of bacterial origin | 1.19E-07 | 1.51E-05 | 8 |
| GO:0050920 | regulation of chemotaxis | 1.42E-07 | 1.60E-05 | 8 |
| GO:0043900 | regulation of multi-organism process | 1.45E-07 | 1.60E-05 | 10 |
| GO:0007204 | positive regulation of cytosolic calcium ion concentration | 2.14E-07 | 2.08E-05 | 9 |
| GO:0071216 | cellular response to biotic stimulus | 2.70E-07 | 2.38E-05 | 8 |
| GO:0032496 | response to lipopolysaccharide | 2.85E-07 | 2.38E-05 | 9 |
| GO:0051607 | defense response to virus | 2.88E-07 | 2.38E-05 | 8 |
| GO:0002237 | response to molecule of bacterial origin | 3.94E-07 | 3.10E-05 | 9 |
| GO:0006874 | cellular calcium ion homeostasis | 4.50E-07 | 3.24E-05 | 10 |
| GO:0051480 | regulation of cytosolic calcium ion concentration | 5.51E-07 | 3.80E-05 | 9 |
| GO:0072503 | cellular divalent inorganic cation homeostasis | 8.80E-07 | 5.14E-05 | 10 |
| GO:0002685 | regulation of leukocyte migration | 1.13E-06 | 5.94E-05 | 7 |
| GO:0070374 | positive regulation of ERK1 and ERK2 cascade | 2.10E-06 | 0.000102121 | 7 |
| GO:0051047 | positive regulation of secretion | 2.47E-06 | 0.000116706 | 9 |
| GO:0007159 | leukocyte cell-cell adhesion | 3.91E-06 | 0.00016573 | 8 |
| GO:0006959 | humoral immune response | 5.85E-06 | 0.000235921 | 8 |
| GO:0030198 | extracellular matrix organization | 7.45E-06 | 0.000293497 | 8 |
| GO:0002791 | regulation of peptide secretion | 8.71E-06 | 0.000327412 | 9 |
| GO:0022407 | regulation of cell-cell adhesion | 1.44E-05 | 0.000518244 | 8 |
| GO:0071356 | cellular response to tumor necrosis factor | 1.53E-05 | 0.000537083 | 7 |
| GO:0070661 | leukocyte proliferation | 1.78E-05 | 0.000611532 | 7 |
| GO:0030099 | myeloid cell differentiation | 1.81E-05 | 0.000611532 | 8 |
| GO:0070372 | regulation of ERK1 and ERK2 cascade | 1.86E-05 | 0.000614382 | 7 |
| GO:0043062 | extracellular structure organization | 2.01E-05 | 0.000643274 | 8 |
| GO:1903037 | regulation of leukocyte cell-cell adhesion | 2.02E-05 | 0.000643274 | 7 |
| GO:0034612 | response to tumor necrosis factor | 2.39E-05 | 0.000745603 | 7 |
| GO:0050863 | regulation of T cell activation | 2.49E-05 | 0.000762365 | 7 |
| GO:0070371 | ERK1 and ERK2 cascade | 2.65E-05 | 0.000781256 | 7 |
| GO:0032103 | positive regulation of response to external stimulus | 2.98E-05 | 0.000835893 | 7 |
| GO:0051235 | maintenance of location | 3.42E-05 | 0.000942312 | 7 |
| GO:0001667 | ameboidal-type cell migration | 3.77E-05 | 0.001006072 | 8 |
| GO:0050708 | regulation of protein secretion | 4.46E-05 | 0.001151543 | 8 |
| GO:0048871 | multicellular organismal homeostasis | 5.40E-05 | 0.00133208 | 8 |
| GO:1903532 | positive regulation of secretion by cell | 0.000112804 | 0.002487706 | 7 |
| GO:0042110 | T cell activation | 0.000284771 | 0.005233455 | 7 |
| GO:1903706 | regulation of hemopoiesis | 0.000328132 | 0.005582784 | 7 |
| GO:0051249 | regulation of lymphocyte activation | 0.000372023 | 0.005942251 | 7 |
| GO:0005125 | cytokine activity | 1.05E-12 | 1.87E-10 | 12 |
| GO:0048018 | receptor ligand activity | 3.52E-12 | 3.15E-10 | 15 |
| GO:0008009 | chemokine activity | 8.42E-11 | 5.03E-09 | 7 |
| GO:0005126 | cytokine receptor binding | 4.35E-10 | 1.95E-08 | 11 |
| GO:0042379 | chemokine receptor binding | 7.36E-10 | 2.63E-08 | 7 |
| GO:0008201 | heparin binding | 5.20E-07 | 1.55E-05 | 7 |
| GO:0001664 | G protein-coupled receptor binding | 1.25E-06 | 3.21E-05 | 8 |
| GO:0005539 | glycosaminoglycan binding | 3.95E-06 | 7.86E-05 | 7 |
| GO:1901681 | sulfur compound binding | 7.04E-06 | 0.000114485 | 7 |
| hsa04657 | IL-17 signaling pathway | 0.001286796 | 0.029918015 | 4 |
| hsa04061 | Viral protein interaction with cytokine and cytokine receptor | 1.28E-09 | 1.19E-07 | 9 |
| hsa04060 | Cytokine-cytokine receptor interaction | 1.44E-08 | 6.68E-07 | 12 |
| hsa04062 | Chemokine signaling pathway | 4.03E-06 | 0.000124908 | 8 |
