# Supplementary material for: A Prognostic Nomogram Combining Immune-Related Gene Signature and Clinical Factors Predicts Survival in Patients With Lung Adenocarcinoma
Source: Front Oncol. 2020 Aug 6;10:1300. doi: 10.3389/fonc.2020.01300 (PMC7424034; doi:10.3389/fonc.2020.01300)
Supplement: Table S2 — Univariate Cox analysis of intersection differentially expressed gene in the TCGA and GEO databases. [file Table_2.DOCX]

**Table S2: Univariate Cox analysis of the common differentially expressed gene in the TCGA and GEO databases.**

| TCGA | | | | GEO (GSE68465) | | | |
| --- | --- | --- | --- | --- | --- | --- | --- |
| Genes | HR | 95% CI | P-value | Genes | HR | 95% CI | P-value |
| BDNF | 1.50 | (1.03-2.19) | 0.034 | CCL19 | 0.42 | 0.23-0.74 | 0.003 |
| **CD79A** | 0.87 | 0.77-0.97 | 0.015 | **CD79A** | 0.38 | 0.18-0.83 | 0.015 |
| CTSG | 0.72 | 0.55-0.95 | 0.021 | LHB | 0.55 | 0.36-0.85 | 0.007 |
| CX3CR1 | 0.70 | 0.54-0.91 | 0.007 | **MAL** | 0.18 | 0.06-0.54 | 0.002 |
| GREM1 | 1.16 | 1.01-1.33 | 0.036 | **MMP12** | 1.83 | 1.06-3.18 | 0.031 |
| ISG15 | 1.17 | 1.03-1.31 | 0.013 | **MS4A1** | 0.48 | 0.25-0.92 | 0.027 |
| LAX1 | 0.72 | 0.57-0.92 | 0.008 | **OAS1** | 4.43 | 1.62-12.07 | 0.004 |
| **MAL** | 0.73 | 0.60-0.89 | 0.002 | S100A2 | 2.08 | 1.14-3.77 | 0.017 |
| **MMP12** | 1.10 | 1.00-1.21 | 0.045 | **WFDC2** | 0.41 | 0.19-0.87 | 0.019 |
| **MS4A1** | 0.76 | 0.64-0.90 | 0.001 |  |  |  |  |
| **OAS1** | 1.22 | 1.04-1.44 | 0.018 |  |  |  |  |
| PTX3 | 1.30 | 1.05-1.62 | 0.015 |  |  |  |  |
| **WFDC2** | 0.83 | 0.75-0.91 | 0.000 |  |  |  |  |
