# Supplementary material for: A Prognostic Nomogram Combining Immune-Related Gene Signature and Clinical Factors Predicts Survival in Patients With Lung Adenocarcinoma
Source: Front Oncol. 2020 Aug 6;10:1300. doi: 10.3389/fonc.2020.01300 (PMC7424034; doi:10.3389/fonc.2020.01300)
Supplement: Table S3 — Gene set variation analysis in the high- and low-risk groups. [file Table_3.DOCX]

**Table S3: Gene set variation analysis in the high- and low-risk groups.**

| Gene sets | logFC | AveExpr | t | P.Value | adj.P.Val | B |
| --- | --- | --- | --- | --- | --- | --- |
| HALLMARK_E2F_TARGETS | -0.31723219 | -0.043315763 | -9.486497337 | 9.40E-20 | 4.70E-18 | 34.02161509 |
| HALLMARK_G2M_CHECKPOINT | -0.249353358 | -0.048285117 | -9.069169764 | 2.67E-18 | 6.67E-17 | 30.72718588 |
| HALLMARK_MYOGENESIS | 0.159117796 | 0.019456319 | 8.499523576 | 2.16E-16 | 2.81E-15 | 26.40414998 |
| HALLMARK_MYC_TARGETS_V1 | -0.256310501 | -0.040608059 | -8.494236865 | 2.25E-16 | 2.81E-15 | 26.36500148 |
| HALLMARK_GLYCOLYSIS | -0.155466988 | -0.016800292 | -8.456413808 | 2.99E-16 | 2.99E-15 | 26.08545117 |
| HALLMARK_MYC_TARGETS_V2 | -0.27566474 | -0.043526567 | -8.161237875 | 2.66E-15 | 2.22E-14 | 23.93625294 |
| HALLMARK_UNFOLDED_PROTEIN_RESPONSE | -0.155625919 | -0.04474051 | -7.828267181 | 2.93E-14 | 2.09E-13 | 21.58240269 |
| HALLMARK_MTORC1_SIGNALING | -0.200177249 | -0.0384517 | -7.528144629 | 2.39E-13 | 1.49E-12 | 19.52671505 |
| HALLMARK_BILE_ACID_METABOLISM | 0.115918564 | 0.027248738 | 7.3671993 | 7.16E-13 | 3.98E-12 | 18.45069879 |
| HALLMARK_PI3K_AKT_MTOR_SIGNALING | -0.097728181 | -0.026078307 | -7.264602268 | 1.43E-12 | 7.14E-12 | 17.77454634 |
| HALLMARK_APICAL_SURFACE | 0.130424156 | 0.019246062 | 6.990394211 | 8.72E-12 | 3.96E-11 | 16.00532377 |
| HALLMARK_KRAS_SIGNALING_DN | 0.072017657 | 0.053950762 | 6.433524088 | 2.90E-10 | 1.21E-09 | 12.58654135 |
| HALLMARK_ANGIOGENESIS | 0.16329125 | 0.051039504 | 5.915866075 | 6.10E-09 | 2.34E-08 | 9.625341977 |
| HALLMARK_IL2_STAT5_SIGNALING | 0.097038516 | -0.006992405 | 5.374391738 | 1.18E-07 | 4.20E-07 | 6.760375002 |
| HALLMARK_DNA_REPAIR | -0.108821348 | -0.050734208 | -5.324078395 | 1.53E-07 | 5.10E-07 | 6.506539278 |
| HALLMARK_ALLOGRAFT_REJECTION | 0.155657461 | -0.00857318 | 5.153591817 | 3.67E-07 | 1.15E-06 | 5.662318855 |
| HALLMARK_PROTEIN_SECRETION | -0.11560936 | -0.04325405 | -5.08519858 | 5.19E-07 | 1.53E-06 | 5.330589606 |
| HALLMARK_MITOTIC_SPINDLE | -0.096167715 | -0.026548197 | -4.888512629 | 1.37E-06 | 3.67E-06 | 4.398948668 |
| HALLMARK_INFLAMMATORY_RESPONSE | 0.130452327 | 0.019455419 | 4.884308255 | 1.40E-06 | 3.67E-06 | 4.379397814 |
| HALLMARK_HEME_METABOLISM | 0.060889897 | -0.008945812 | 4.735318128 | 2.85E-06 | 7.11E-06 | 3.696478005 |
| HALLMARK_IL6_JAK_STAT3_SIGNALING | 0.13038383 | 0.018852561 | 4.586017771 | 5.70E-06 | 1.36E-05 | 3.031567835 |
| HALLMARK_UV_RESPONSE_UP | -0.053876828 | -0.014673966 | -4.388809534 | 1.39E-05 | 3.16E-05 | 2.183387104 |
| HALLMARK_HEDGEHOG_SIGNALING | 0.099001575 | 0.027294162 | 3.746870244 | 0.00019973 | 0.000434196 | -0.336299698 |
| HALLMARK_ADIPOGENESIS | -0.05571708 | -0.029322569 | -3.446470738 | 0.000615363 | 0.00124912 | -1.386297158 |
| HALLMARK_INTERFERON_GAMMA_RESPONSE | 0.108825665 | -0.002473901 | 3.440704544 | 0.000628316 | 0.00124912 | -1.405635407 |
| HALLMARK_KRAS_SIGNALING_UP | 0.057773603 | 0.009545314 | 3.431490897 | 0.000649542 | 0.00124912 | -1.436471357 |
| HALLMARK_UV_RESPONSE_DN | 0.082108914 | -0.012278205 | 3.276641301 | 0.001122795 | 0.00207925 | -1.942888193 |
| HALLMARK_WNT_BETA_CATENIN_SIGNALING | 0.073757063 | -0.00019393 | 3.131086159 | 0.001842592 | 0.003290343 | -2.398466343 |
| HALLMARK_NOTCH_SIGNALING | 0.067313556 | -0.018101463 | 2.998815809 | 0.002844075 | 0.004903577 | -2.795188977 |
| HALLMARK_ESTROGEN_RESPONSE_EARLY | 0.044408414 | -0.005982567 | 2.734900074 | 0.006459446 | 0.010765743 | -3.537285742 |
| HALLMARK_OXIDATIVE_PHOSPHORYLATION | -0.080940451 | -0.055707602 | -2.432507585 | 0.015341225 | 0.024743912 | -4.305909399 |
| HALLMARK_XENOBIOTIC_METABOLISM | 0.034729244 | 0.013833074 | 2.257990755 | 0.024372624 | 0.038082225 | -4.709502403 |
| HALLMARK_FATTY_ACID_METABOLISM | 0.044195338 | -0.02212014 | 2.184426107 | 0.029390398 | 0.044530906 | -4.87081416 |
| HALLMARK_PANCREAS_BETA_CELLS | 0.04500396 | 0.067255658 | 1.960723011 | 0.050460979 | 0.074143479 | -5.329090329 |
| HALLMARK_PEROXISOME | -0.036033058 | -0.012058448 | -1.948571737 | 0.051900435 | 0.074143479 | -5.35258955 |
| HALLMARK_ESTROGEN_RESPONSE_LATE | 0.031016104 | 0.009576406 | 1.837385692 | 0.066740755 | 0.092695493 | -5.560923659 |
| HALLMARK_SPERMATOGENESIS | -0.022838782 | 0.037403099 | -1.809421408 | 0.070980425 | 0.095919493 | -5.611421809 |
| HALLMARK_INTERFERON_ALPHA_RESPONSE | 0.052036287 | -0.020941208 | 1.660026579 | 0.097530319 | 0.128329367 | -5.868237498 |
| HALLMARK_REACTIVE_OXYGEN_SPECIES_PATHWAY | 0.035096511 | -0.022891112 | 1.636493481 | 0.102359799 | 0.131230512 | -5.90669815 |
| HALLMARK_APOPTOSIS | 0.018447575 | -0.015380393 | 1.064817571 | 0.287467704 | 0.35933463 | -6.673538375 |
| HALLMARK_EPITHELIAL_MESENCHYMAL_TRANSITION | -0.030484787 | -0.003168469 | -0.980665952 | 0.3272274 | 0.39831616 | -6.759159056 |
| HALLMARK_TNFA_SIGNALING_VIA_NFKB | -0.024519901 | -0.010806451 | -0.965842586 | 0.334585575 | 0.39831616 | -6.773514285 |
| HALLMARK_ANDROGEN_RESPONSE | 0.01568535 | -0.024003331 | 0.85099371 | 0.395176456 | 0.459507506 | -6.877351266 |
| HALLMARK_TGF_BETA_SIGNALING | -0.018479648 | -0.021366767 | -0.777406133 | 0.437283473 | 0.496913038 | -6.937002154 |
| HALLMARK_APICAL_JUNCTION | 0.010785437 | 0.010951338 | 0.615293408 | 0.538638336 | 0.59848704 | -7.049430339 |
| HALLMARK_CHOLESTEROL_HOMEOSTASIS | 0.009398533 | -0.018299849 | 0.440110891 | 0.660045231 | 0.717440468 | -7.141536301 |
| HALLMARK_HYPOXIA | -0.006319902 | 0.019552938 | -0.397305187 | 0.691310409 | 0.735436605 | -7.159397741 |
| HALLMARK_COMPLEMENT | 0.005458409 | 0.005485449 | 0.256320424 | 0.797807884 | 0.831049879 | -7.205320727 |
| HALLMARK_P53_PATHWAY | 0.000988387 | -0.024435755 | 0.076639449 | 0.93894075 | 0.958102806 | -7.235139419 |
| HALLMARK_COAGULATION | 0.000629654 | 0.036859854 | 0.035978904 | 0.971313407 | 0.971313407 | -7.237421888 |
| GO_INOSITOL_TRISPHOSPHATE_PHOSPHATASE_ACTIVITY | -0.228403827 | -0.025140847 | -10.25315379 | 1.58E-22 | 1.58E-18 | 40.39560924 |
| GO_INOSITOL_POLYPHOSPHATE_5_PHOSPHATASE_ACTIVITY | -0.225470817 | -0.028834971 | -9.633909876 | 2.85E-20 | 2.84E-16 | 35.30905563 |
| GO_IMMUNOGLOBULIN_COMPLEX | -0.222266448 | -0.011084954 | -8.243625599 | 1.47E-15 | 1.46E-11 | 24.71019078 |
| GO_NEGATIVE_REGULATION_OF_CELL_CHEMOTAXIS_TO_FIBROBLAST_GROWTH_FACTOR | -0.23192339 | -0.015698859 | -6.498086262 | 1.96E-10 | 1.93E-06 | 13.2328533 |
| GO_ANTIGEN_PROCESSING_AND_PRESENTATION_ENDOGENOUS_LIPID_ANTIGEN_VIA_MHC_CLASS_IB | -0.201673274 | -0.005804241 | -6.365125817 | 4.40E-10 | 4.33E-06 | 12.45067222 |
| GO_LIPID_ANTIGEN_BINDING | -0.201673274 | -0.005804241 | -6.365125817 | 4.40E-10 | 4.33E-06 | 12.45067222 |
| GO_ALPHA_BETA_T_CELL_RECEPTOR_COMPLEX | -0.202733344 | -0.016053859 | -5.776589231 | 1.34E-08 | 0.000130296 | 9.155433608 |
