# Supplementary material for: A Prognostic Nomogram Combining Immune-Related Gene Signature and Clinical Factors Predicts Survival in Patients With Lung Adenocarcinoma
Source: Front Oncol. 2020 Aug 6;10:1300. doi: 10.3389/fonc.2020.01300 (PMC7424034; doi:10.3389/fonc.2020.01300)
Supplement: Table S4 — 45 transcription factors associated with the four immune-related genes. [file Table_4.DOCX]

**Table S4: 45 transcription factors associated with the four immune-related genes.**

| ImmuneGene | TF | Cor | P-value | Regulation |
| --- | --- | --- | --- | --- |
| MAL | CBX7 | 0.404325485 | 4.35E-21 | postive |
|  | LYL1 | 0.341873531 | 3.73E-15 | postive |
|  | STAT5A | 0.34011675 | 5.26E-15 | postive |
|  | TCF21 | 0.332787624 | 2.15E-14 | postive |
| MS4A1 | CBX7 | 0.4886149 | 2.30E-31 | postive |
|  | CIITA | 0.525531991 | 7.55E-37 | postive |
|  | ETS1 | 0.455586653 | 5.42E-27 | postive |
|  | EZH1 | 0.338827282 | 6.75E-15 | postive |
|  | FLI1 | 0.554584038 | 1.15E-41 | postive |
|  | FOXP3 | 0.476215658 | 1.15E-29 | postive |
|  | IKZF1 | 0.73503942 | 4.43E-86 | postive |
|  | IRF4 | 0.446226778 | 7.74E-26 | postive |
|  | KAT2B | 0.35997089 | 9.63E-17 | postive |
|  | LYL1 | 0.543494046 | 9.03E-40 | postive |
|  | MEF2C | 0.353587371 | 3.60E-16 | postive |
|  | NFATC1 | 0.47062352 | 6.35E-29 | postive |
|  | PAX5 | 0.595612261 | 2.50E-49 | postive |
|  | PRDM1 | 0.439769732 | 4.62E-25 | postive |
|  | SPIB | 0.448248328 | 4.39E-26 | postive |
|  | STAT4 | 0.365459582 | 3.03E-17 | postive |
|  | STAT5A | 0.509219414 | 2.42E-34 | postive |
|  | STAT5B | 0.359094141 | 1.16E-16 | postive |
|  | TCF7 | 0.653541708 | 3.13E-62 | postive |
| OAS1 | PML | 0.328053653 | 5.21E-14 | postive |
|  | STAT1 | 0.432349611 | 3.44E-24 | postive |
|  | STAT2 | 0.344602156 | 2.18E-15 | postive |
| WFDC2 | BACH1 | -0.383582973 | 5.67E-19 | negative |
|  | BRCA1 | -0.392077352 | 8.04E-20 | negative |
|  | CENPA | -0.343997346 | 2.46E-15 | negative |
|  | CHD7 | -0.306753639 | 2.35E-12 | negative |
|  | E2F7 | -0.300389588 | 6.93E-12 | negative |
|  | FOXA2 | 0.322674299 | 1.40E-13 | postive |
|  | FOXM1 | -0.420497052 | 7.65E-23 | negative |
|  | KDM5A | -0.323547408 | 1.20E-13 | negative |
|  | LIN9 | -0.321457756 | 1.75E-13 | negative |
|  | MYC | -0.332833606 | 2.13E-14 | negative |
|  | NCAPG | -0.415548707 | 2.70E-22 | negative |
|  | POLR3A | -0.354992516 | 2.70E-16 | negative |
|  | POLR3G | -0.378119118 | 1.93E-18 | negative |
|  | PRKDC | -0.414896959 | 3.18E-22 | negative |
|  | SMC3 | -0.345813088 | 1.72E-15 | negative |
|  | SSRP1 | -0.305998154 | 2.68E-12 | negative |
|  | TEAD1 | -0.331137684 | 2.93E-14 | negative |
|  | TEAD4 | -0.423900739 | 3.18E-23 | negative |
|  | TTF2 | -0.334438111 | 1.57E-14 | negative |
