# Supplementary material for: A Prognostic Nomogram Combining Immune-Related Gene Signature and Clinical Factors Predicts Survival in Patients With Lung Adenocarcinoma
Source: Front Oncol. 2020 Aug 6;10:1300. doi: 10.3389/fonc.2020.01300 (PMC7424034; doi:10.3389/fonc.2020.01300)
Supplement: Table S5 — The relationship between four immune-related genes and clinical features. [file Table_5.DOCX]

**Table S5:**  **Relationship between four immune-related genes and clinical features.**

| Variables | MAL | MS4A1 | OAS1 | WFDC2 |
| --- | --- | --- | --- | --- |
|  | Cor (P-value) | Cor (P-value) | Cor (P-value) | Cor (P-value) |
| Age | -0.003 (0.998) | -2.265 (0.024) | 0.47 (0.638) | -0.484 (0.628) |
| Sex | 2.753 (0.006) | 3.303 (0.001) | -0.042 (0.966) | 1.782 (0.075) |
| T_stage | 2.836 (0.006) | 5.023 (2.225e-06) | -0.667 (0.507) | 1.895 (0.062) |
| N_stage | 0.934 (0.351) | 2.683 (0.008) | -3.288 (0.001) | 1.375 (0.170) |
| TNM_Stage | 1.563 (0.120) | 3.554 (4.812e-04) | -1.993 (0.048) | 2.07 (0.040) |
| Riskscores | -13.038 (5.049e-33) | -13.546 (2.936e-34) | 7.629 (1.467e-13) | -11.964 (1.26e-28) |
